# 25 years of applying the 3Rs principles in safety pharmacology: success stories and future perspectives

**DOI:** 10.3389/fphys.2026.1813708

**Published:** 2026-04-27

**Authors:** Katayoun Derakhchan, Kimberly Rockley, Matthias Gossmann, Raafat Fares, Eric Delpy, Yasunari Kanda, Elisa Passini, Colleen M. Pike, Janette Turner, Annie Delaunois

**Affiliations:** 1 Pioneering Medicines at Flagship Pioneering, Cambridge, MA, United States; 2 Apconix, Cheshire, United Kingdom; 3 innoVitro GmbH, Jülich, Germany; 4 ERBC France, Baugy, France; 5 Biotrial Pharmacology, Rennes, France; 6 Division of Pharmacology, National Institute of Health Sciences (NIHS), Kawasaki, Japan; 7 National Centre for the Replacement, Refinement and Reduction of Animals in Research (NC3Rs), London, United Kingdom; 8 Humane World for Animals, Washington, DC, United States; 9 UCB, Braine-l’Alleud, Belgium

**Keywords:** 3Rs principles, animal welfare, CiPA, NAMs, reduction, refinement, replacement, safety pharmacology

## Abstract

Safety pharmacology is a relatively young discipline dedicated to identifying adverse pharmacodynamic effects of new drugs that may impact human safety. Since the publication of the ICH S7A guideline in 2000, the field has made significant efforts in implementing the 3Rs principles—Replacement, Reduction, and Refinement of animal use. This review provides a comprehensive overview of key milestones and innovations that have shaped the application of the 3Rs in safety pharmacology over the past 25 years. Typical examples are the use of non-invasive jacket telemetry for real-time cardiovascular monitoring in conscious freely moving animals and the introduction of the CiPA (Comprehensive in Vitro Proarrhythmia Assessment) paradigm in 2014. The review explores 3Rs-driven advances across core organ systems—cardiovascular, central nervous, and respiratory—as well as supplemental systems including gastrointestinal and renal. Furthermore, it examines the evolving global regulatory landscape and the emergence of new approach methodologies (NAMs), such as artificial intelligence tools and complex microphysiological systems (MPS), and their integration into drug development pipelines. Finally, the review discusses future directions and potential challenges in achieving broader adoption of 3Rs-compliant strategies and NAMs in safety pharmacology.

## 1 The 3Rs principles: definition and history

The principles of 3Rs (Replacement, Reduction and Refinement) were originally developed by William Russell and Rex Burch in 1959, in their book “The Principles of Humane Experimental Technique” (Russell and Burch 1959). They proposed that—if animals were to be used in experiments —every effort should be made to Replace them with non-sentient alternatives, to Reduce to a minimum the number of animals used, and to Refine experiments which used animals so that they caused the minimum pain and distress. Together, these principles promote a balance between scientific advancement and ethical responsibility and provide a framework to promote more humane and responsible animal research.

Over the decades, the 3Rs principles have been embedded in national and international legislation and regulations on the use of animals in scientific procedures, as well as in the policies of organizations funding or conducting animal research. To advance the 3Rs principles in practice, various 3Rs centers have emerged worldwide, for example: the Center for Alternatives in Animal Testing (CAAT), Johns Hopkins University, in 1981; the UK National Centre for the Replacement, Refinement and Reduction of Animals in Research (NC3Rs) in 2004; the North American 3Rs Collaborative (NA3RsC) in 2017; the Swiss 3R Competence Centre (3RCC) in 2018; the French Center for the 3Rs (FC3Rs) in 2021, and many more. The International Cooperation on Alternative Test Methods (ICATM) was established in 2009 to advance the global implementation of the 3Rs by supporting the development, scientific validation, and regulatory uptake of human−relevant new approach methodologies (NAMs) for safety assessment. Within the ICATM framework, partners including the Interagency Coordinating Committee on the Validation of Alternative Methods (ICCVAM, United States), the European Centre for the Validation of Alternative Methods (ECVAM, European Union (EU)), the Japanese Center for the Validation of Alternative Methods (JaCVAM, Japan) and other national and regional validation centers, coordinate activities to harmonize validation approaches, share expertise, and promote convergence in the international regulatory acceptance of NAMs and promote convergence in the regulatory acceptance of NAMs across regions.

Whilst the 3Rs principles themselves have remained the same since their original publication, there have been changes in their interpretation and definition (Tannenbaum and Bennett, 2015). The NC3Rs re-defined the 3Rs principles to make them more reflective of contemporary scientific practice and developments (NC3Rs, 2025), as shown in **Table 1**.

**Table 1:** Basic and updated definitions of the 3Rs.

R principle	Basic (Russell and Burch, 1959)	Updated (NC3Rs, 2025)
Replacement	Avoiding or replacing the use of animals in areas where they otherwise would have been used.	Accelerating the development and use of predictive and robust models and tools, based on the latest science and technologies, to replace the use of animals in addressing important research questions where they would have otherwise been used.
Reduction	Minimising the number of animals used consistent with scientific aims.	Appropriately designed and analysed animal experiments that are robust and reproducible, and add to the knowledge base.
Refinement	Minimising the pain, suffering, distress or lasting harm that research animals might experience.	Advancing laboratory animal welfare by exploiting the latest *in vivo* technologies to minimise pain, suffering and distress and improve understanding of the impact of welfare on scientific outcomes.

In recent years, the remarkable advances *in vitro* and *in silico* techniques have accelerated the development of NAMs, such as organoids or virtual tissue models. These can provide improved and more human-relevant safety assessments for (agro) chemicals and drugs, reducing the reliance on traditional animal toxicity tests studies and significantly advancing the 3Rs principle of Replacement (Sewell et al., 2024).

Animal use is reported in many regions and continues to decline overall (e.g., an 8.2% reduction in the EU in 2023 vs 2022 (European Animal Research Association (EARA, 2026a)). However, while these data provide a useful high-level overview, they lack the granularity needed to explain the drivers of change or quantify the impact of specific 3Rs approaches, study-design innovations, or NAM adoption. Current reporting rarely estimates animals avoided through 3Rs interventions or links reductions to regulatory flexibilities (e.g., study waivers), alternative designs, or updated guidance. Consequently, stakeholders cannot robustly attribute reductions to specific refinements (e.g., telemetry crossover designs or TQT waivers following the ICH E14/S7B revision). Strengthening the evidence base therefore requires continued statistics alongside greater transparency on study intent, regulatory decision-making, and drivers of change. National transparency agreements on animal research such as those coordinated by the EARA (e.g., the UK Concordat on Openness in Animal Research or the Australian Transparency Agreement on Animal Research), now established across ten countries, provide a practical framework to support openness, contextual reporting, and shared learning beyond headline numbers (EARA, 2026b).

## 2 Evolution of regulatory framework on animal testing across the world

**Figure 1:**
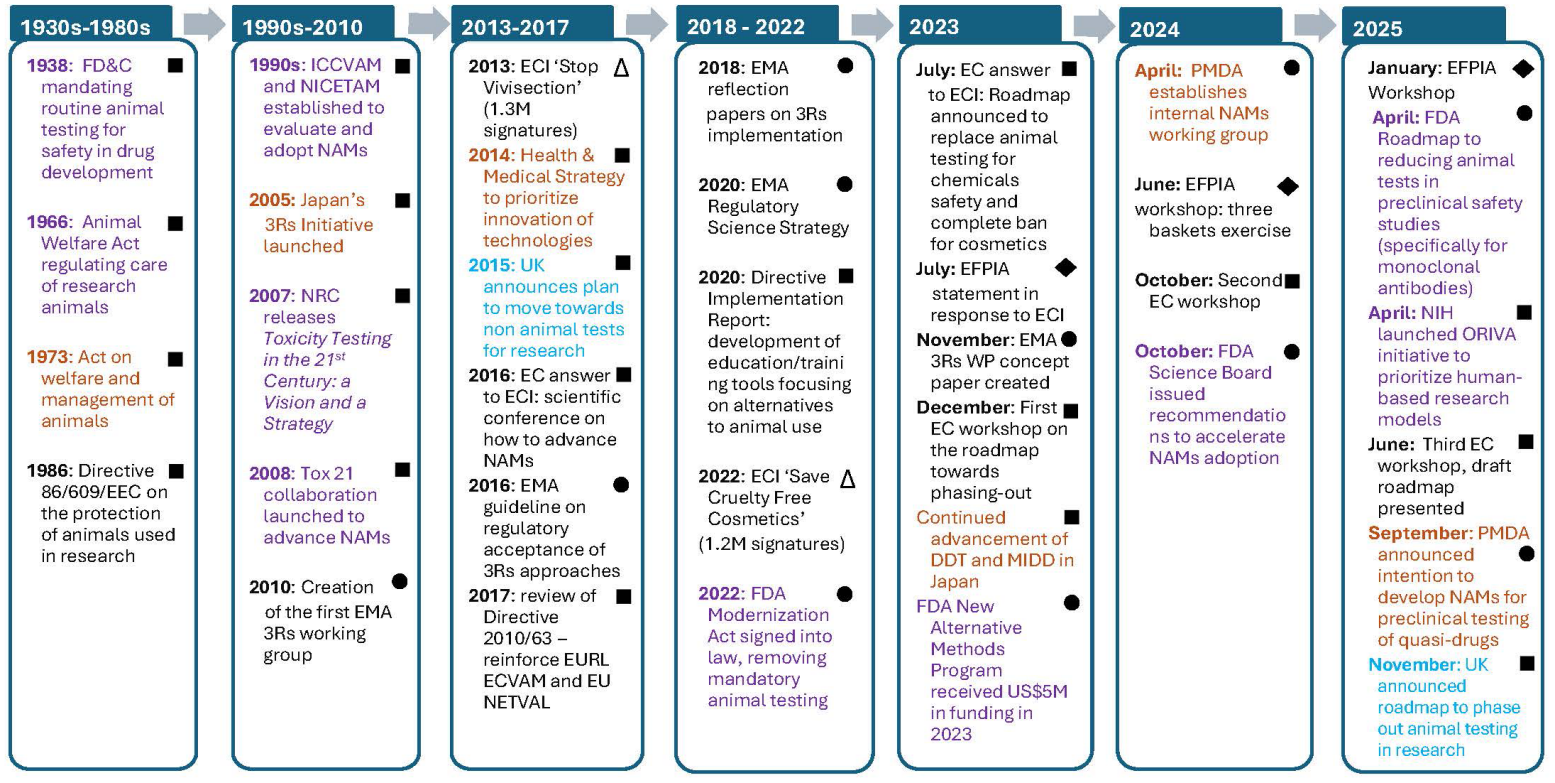
Key actions that drove the evolution of the regulatory framework on animal testing across different regions of the world (black: Europe; purple: USA; amber: Japan; blue: UK). Squares: governmental actions; circles: drug agencies actions; diamonds: industry actions; triangles: public actions.

Global regulatory systems have progressively shifted toward reducing and replacing animal use in scientific research. Although the pace and drivers of change differ between regions of the world, there is now a shared commitment to integrating the 3Rs and advancing NAMs (**Table 2**). Regulatory frameworks have evolved in response to scientific and technological innovation, as well as to societal expectations and political pressure (**Figure 1**). The major milestones shaping this transition in the European Union (EU), the United Kingdom (UK), the United States (USA), and Japan are as follows.

**Table 2:** Comparison of main characteristics driving the regulatory shift for phasing out of animal testing and 3Rs/NAMs implementation across different regions of the world.

Characteristics	European Union	United Kingdom	United States	Japan
Legal foundation for 3Rs/NAMs in research	Strongest formal legal mandate (Directive 2010/63/EU)	Initially aligned with EU; Post-Brexit strategy reaffirmed commitment	Historically required animal testing, legal requirements relaxed in 2022	Regulatory encouragement rather than explicit legal mandates
Key institutions	EURL-ECVAM, EMA, EC	NC3Rs, UKCVAM, DSTI	FDA, NICEATM, ICCVAM, NIH	JaCVAM/NIHS, PMDA, MHLW
Public/political pressure	High (multiple ECIs influencing policy)	Growing public interest, strong policy response post-Brexit	Moderate (scientific drivers rather than public pressure)	Lower public pressure; change driven by national innovation strategies
Strategic roadmaps	EU roadmap to phase out animal testing in chemical safety (expected 2026)	Replacing animals in science strategy (2025)	FDA NAM roadmap (2025)	Health and Medical Strategy (revised every 5 years)
Scientific leadership areas	Validation frameworks, regulatory harmonization, *in vitro* toxicity tests (ECVAM)	Adoption frameworks, national-scale coordination	Mechanistic toxicology, high-throughput screening, legal reform	iPSC-based safety testing, organ-on-chip platforms
Funding mechanisms	EU research programs and joint initiatives (IHI, Horizon 2020,…)	Government funding	Federal funding	AMED funding and government funding
Regulatory acceptance of NAMs	Gradually expanding, defined pathways under EMA	Growing but requires stronger validation to meet regulatory needs	Improving rapidly through FDA Modernization Act and guidance efforts	Advancing through DDT qualification and PMDA engagement
Primary challenges	Harmonizing acceptance across Member states; accelerating validation, defining context-of-use	Regulatory confidence, scaling NAMs infrastructure	Context-of-use clarity, validation standards, cross-agency consistency	International alignment, expanding validated NAM applications

### 2.1 European Union

The EU has been a global leader in embedding the 3Rs into legislation. Directive 2010/63/EU, which replaced Directive 86/609/EEC, introduced more stringent and transparent measures on the protection of animals used for scientific purposes and made the application of the 3Rs legally mandatory across all member states. Long before this Directive, the European Commission (EC) had already recognized the need for alternatives by establishing ECVAM to coordinate the effort across the Union. Since its creation, more than 40 alternative methods mostly used in toxicology have been developed and validated by ECVAM, who became the EU Reference Laboratory (EURL-ECVAM) in the field.

Public pressure has been a significant catalyst of paradigm change. EU citizen initiatives, such as the 2013 ‘stop vivisection’ petition and the 2022 ‘Europe without animal testing’ initiative, prompted increasing political attention. In response, the EC organized a scientific conference on non-animal approaches, reinforced the role of EURL-ECVAM, and also committed to developing a roadmap to phase out animal testing for chemical safety assessments, expected in 2026.

In parallel to these efforts, the European Medicines Agency (EMA) have issued guidelines and reflection papers to facilitate acceptance of 3Rs approaches. Collaboration across stakeholders (industry, academia, regulators, non-profit organizations, etc.) has intensified, with various workshops organized by the EC, EMA 3Rs Working Party, and the European Federation of Pharmaceutical Industries and Associations (EFPIA). Of note, the EFPIA proposed a structured methodology to categorize all animal studies in three baskets to facilitate the prioritization of NAMs development and validation (EFPIA, 2025).

### 2.2 United Kingdom

While part of the EU, until 31 January 2020, the UK was bound by Directive 2010/63/EU and fully applied the 3Rs principles in animal experimentation.

In 2015, Innovate UK on behalf of a group of six funding UK bodies (including the Medical Research Council (MRC) and the NC3Rs) published a non-animal technologies roadmap (Innovate UK, 2015). This roadmap is intended to set a strategic vision for non-animal technologies in the UK and guide the efforts of those working in this area towards 2030. Following Brexit, the UK renewed its independent commitment to NAMs. In November 2025, the Department of Science, Technology and Innovation (DSTI) released the strategy “Replacing animals in science” (Government of the United Kingdom, 2025) which, like the EFPIA “three baskets” approach, facilitates the prioritization of NAMs development and validation. The DSIT acknowledged the increasing use of alternative methods but also highlighted continued regulatory and scientific barriers to broader adoption. With newer advances in technology— particularly AI and genomics, but also organoid and 3D cell systems—it was felt that a path to changing the reliance on animals in science was possible.

To accelerate progress, the UK committed £75 million in funding, comprising of £15.9M for “human *in vitro* models” and £60M for the creation of a preclinical translational models’ hub and a UK Centre for the Validation of Alternative Methods (UKCVAM). In addition, this roadmap contains five key commitments and 26 strategy commitments, including a publicly available dashboard of progress against key deliverables.

### 2.3 United States

The evolution of NAMs in the United States builds upon a regulatory foundation established by the Animal Welfare Act (AWA) and the Federal Food, Drug, and Cosmetic (FD&C) Act, which historically mandated animal testing to support the safety of drugs, chemicals, and biologics. Momentum to develop and validate alternatives increased in the late 1990s with the establishment of the National Toxicology Program’s Interagency Center for the Evaluation of Alternative Toxicological Methods (NICEATM) which, in coordination with the ICCVAM, expanded cross-agency coordination and scientific review of non-animal test methods.

A major conceptual shift occurred with the 2007 National Research Council report “Toxicity Testing in the 21st Century: A Vision and a Strategy”, advocating mechanistic, human-relevant testing systems (National Research Council of the National Academies of Science, 2007). This led to the launch of the Tox21 program, a collaboration among the EPA, FDA, NIEHS, and NIH’s National Center for Advancing Translational Sciences (NCATS)—, which significantly advanced high-throughput screening tools and computational toxicology (Thomas et al., 2018).

The early 2020s marked a turning point in the formal recognition and integration of NAMs within the U.S. regulatory framework. In 2022, the FDA Modernization Act 2.0 removed the legal mandate requiring animal testing for drug entering clinical trials. That same year, the FDA Science Board introduced the New Alternative Methods Program to advance the agency’s ability to implement NAMs into regulatory practice and expanded qualification pathways, such as the Drug Development Tool and ISTAND programs, providing guidance to developers of alternative approaches. In 2023, this program received $5 million in dedicated funding to advance research, infrastructure, and regulatory implementation efforts (U.S. Food and Drug Administration (FDA), 2023). Building on this momentum, the FDA Science Board in 2024 issued a set of recommendations aimed at accelerating the regulatory implementation of NAMs (FDA, 2024).

In 2025, the FDA published a roadmap for reducing reliance on animal testing in the development of monoclonal antibodies and other drugs (FDA, 2025), followed by publications detailing CDER’s experience evaluating NAMs and offering recommendations to strengthen their regulatory role (Yao et al., 2025). In parallel, the NIH created the Office of Research Innovation, Validation, and Application, to promote human-based research models and coordinate NAM-related efforts across agencies (NIH, 2025).

### 2.4 Japan

In Japan, the adoption of NAMs in pharmaceutical development has accelerated in response to global trends aimed at reducing animal testing, improving translational predictivity, and advancing regulatory science. The establishment of JaCVAM in 2005 within the National Institute of Health Sciences (NIHS) marked the beginning of coordinated validation efforts for regulatory acceptance of alternative methods. NAMs approaches, including emerging technologies such as organ-on-a-chip and assays derived from induced pluripotent stem cells (iPSC), have become increasingly significant in the context of Japan’s Health and Medical Strategy—first introduced in 2014 and revised every five years—which prioritizes the promotion of innovative medical technologies, the acceleration of drug development, and the strengthening of international competitiveness through regulatory science.

In recent years, Japan actively promotes the advancement of Drug Development Tools (DDTs), a regulatory framework designed to qualify innovative methodologies, including biomarkers, clinical outcome assessments, and NAM-based technologies. The Pharmaceuticals and Medical Devices Agency (PMDA) has strengthened its engagement in NAM implementation by establishing internal working groups dedicated to regulatory science and alternative methodologies. PMDA has further expressed its intention to promote the regulatory application of NAMs in preclinical safety assessment, including for quasi-drugs, in alignment with international regulatory developments. Ongoing efforts emphasize harmonization of DDT qualification processes and promotion of international acceptance of NAM-derived data, thereby ensuring their effective integration into global drug development practices.

Japan is internationally recognized for leadership in human iPSC-based safety testing, with significant support from the Japan Agency for Medical Research and Development (AMED), which provides dedicated funding for medical innovation and regulatory science. These models have contributed to advances in cardiotoxicity testing and predictive modeling of drug-induced QT prolongation and arrhythmogenic risk.

Another area of rapid advancement has been the development of organ-on-a-chip and microphysiological systems (MPS). Japanese consortia have engineered platforms that recreate the functional microenvironments of critical human organs, including the liver, kidney, and central nervous system. These technologies enable more accurate evaluation of drug metabolism and toxicity under physiologically relevant conditions, thereby enhancing the translational value of preclinical research.

Overall, Japan’s regulatory science reflects sustained commitment to NAMs, strong national strategic policies and alignment with international scientific and regulatory developments.

## 3 3Rs in safety pharmacology

As initially defined by Pugsley et al (Pugsley et al., 2008), safety pharmacology applies core pharmacological principles within a regulatory framework to generate data that support the risk-benefit evaluation of new therapeutic candidates.

The International Council for Harmonisation (ICH) S7A guideline (International Council for Harmonisation (ICH), 2000) provides the general principles and recommendations for safety pharmacology studies and defines them as “those studies that investigate the potential undesirable pharmacodynamic effects of a substance on physiological functions in relation to exposure in the therapeutic range and above”. The safety pharmacology core battery aims to assess the impact of a test substance on vital physiological functions. This typically includes the cardiovascular, respiratory, and central nervous systems. In certain cases, the core battery may be expanded to investigate potential adverse pharmacodynamic effects on other organ system functions not initially covered, such as the renal and gastrointestinal (GI) systems.

Although ICH S7A guideline explicitly supports the use of non-animal methods where feasible, safety assessment in animals forms an important part of this process, in particular to characterize the pharmacodynamic/pharmacokinetic and the dose-response relationships of the adverse effect observed. Therefore, safety pharmacology packages almost always include *in vivo* assays in both rodents and non-rodent species.

Over the last 25 years, the field has made significant efforts in implementing the 3Rs principles in safety pharmacology (**Table 3**). Regulatory agencies are likewise pushing in the same direction, provided that alternative methods are scientifically validated and predictive of human outcomes. There are growing calls for revision of the ICH S7A guidance to better reflect the latest scientific and technological advancements by considering human-relevant NAMs and reducing unnecessary animal use (Valentin and Leishman, 2023, 2025). The next sections of this review provide examples of 3Rs application for the three vital systems assessed by the core battery of safety pharmacology studies (cardiovascular, central nervous system (CNS) and respiratory) as well as for the renal and GI systems.

**Table 3:** Examples of 3Rs principles application in safety pharmacology.

Organ/system	Model/assay	Assay category	‘R’ principles	Context of use	Key references
Cardiovascular	Electrophysiology simulation in virtual rabbit Purkinje fibers	In silico	Replace, reduce	Exploratory	(Mohr et al., 2022)
Cardiovascular	Electrophysiology and contractility simulation in virtual human cardiac models	In silico	Replace, reduce	Regulatory (ICH S7B follow-up assay)	(Muszkiewicz et al., 2016; Li et al., 2019; Passini et al., 2019; Llopis-Lorente et al., 2020; Trovato et al., 2022; Holmes et al., 2025)
Cardiovascular	Ion channel patch clamp assays on transfected mammalian cells (HEK293, CHO,…)	*In vitro*	Replace, reduce	Exploratory, Regulatory (ICH E14/S7B Q&As)	(Colatsky et al., 2016; Crumb et al., 2016)
Cardiovascular	Electrophysiology, contractility, and calcium flux in human iPSC-cardiomyocytes	*In vitro*	Replace, reduce	Exploratory Regulatory (ICH E14/S7B Q&As)	(Nakamura et al., 2014; Ando et al., 2017; Blinova et al., 2017)
Cardiovascular	Cardiac organoids, EHTs, heart-on-chip	*In vitro*	Replace, reduce	Exploratory	(Goßmann et al., 2016, 2020; Lickiss et al., 2022, 2024)
Cardiovascular	Implanted telemetry in freely moving animals (ECG, blood pressure, left ventricular pressure)	*In vivo*	Reduce, Refine	Regulatory (ICH E14/S7B Q&As)	(Bétat et al., 2024; Rossman et al., 2023; Leishman et al., 2023; Komatsu et al., 2019)
Cardiovascular, respiratory, CNS	Jacketed telemetry in freely moving animals (JET-ECG, JET-BP, JET-RIP, DECRO^®^ jacket)	*In vivo*	Reduce, Refine	Regulatory (ICH S7B, M3, S6)	(Vargas et al., 2010; Derakhchan et al., 2011b, 2011b; Fares et al., 2020, 2022; Flenet et al., 2020; Fares and Champéroux, 2023)
Cardiovascular	Stroke volume modeling and additional hemodynamic parameters in telemetered large animals	In silico/*in vivo*	Reduce, refine	Exploratory	(Hotek et al., 2023; Champeroux et al., 2024)
CNS	Ca^2+^ oscillation in 2D or 3D hiPSC-derived neuronal cultures	*In vitro*	Replace, reduce	Exploratory	(Imredy et al., 2023; Lu et al., 2023)
CNS	Electrical firing on MEA in hiPSC neuronal cultures to assess	*In vitro*	Replace, reduce	Exploratory	(Grainger et al., 2018; Shirakawa and Suzuki, 2020; Tukker et al., 2020; Rockley et al., 2023; Zhai et al., 2023)
CNS	Electrical firing on MEA in primary rodent cortical neuronal cultures	*In vitro*	Reduce	Exploratory	(Bradley et al., 2018; Kreir et al., 2018; Tukker et al., 2020)
CNS	Automated electrophysiological ion channel assays using recombinant cell lines	*In vitro*	Replace, reduce	Exploratory	(Delaunois et al., 2020; Rockley et al., 2023; Cohen et al., 2025)
CNS	3D brain-on-chip models	*In vitro*	Replace	Exploratory	(Fan et al., 2022; Kim and Chang, 2023; Giorgi et al., 2024)
CNS	Smart cages for behavioral assessment	*In vivo*	Refine	Exploratory	(Chen et al., 2023; Chindemi et al., 2025)
CNS	Implanted telemetry in freely moving animals (EEG, EMG recordings)	*In vivo*	Refine, reduce	Exploratory	(Bassett et al., 2014; Delaunois et al., 2020)
CNS	Non-invasive seizure detection by accelerometry or UHD video monitoring	*In vivo*	Refine	Exploratory	(Diaz-Arce et al., 2022; Sabnis et al., 2025)
Respiratory	ALI cultures, lung organoids, precision cut lung slices, lung-on-chip	*In vitro*	Replace	Exploratory	(Miller et al., 2019; Wang et al., 2025)
Gastrointestinal	Ingestable pills measuring transit time, pH, temperature (SmartPill™, Bravo™,…)	*In vivo*	Refine	Exploratory	(Rudholm et al., 2008; Zhou et al., 2008; Kook et al., 2014; Warrit et al., 2017; Koziolek et al., 2019; Knibbe et al., 2023)
Gastrointestinal	2D/3D models: human cell lines/primary cells in transwells, organoids, gut-on-chip	*In vitro*	Replace	Exploratory Regulatory (ICH S7A)	(Peters et al., 2019; Pike et al., 2025)
Renal	2D/3D models, kidney-on-a-chip	*In vitro*	Replace	Exploratory	(Huang et al., 2024; Guimaraes et al., 2025)
Renal	Urine collection by hydrophobic sand in home cage	*In vivo*	Refine	Exploratory	(Hoffman et al., 2018)
General	Microsampling	*In vivo*	Refine, reduce	Exploratory, regulatory	(Hopper, 2016)
General	Automated/smart sampling techniques (blood, urine)	*In vivo*	Refine	Exploratory, regulatory	(Han et al., 2013)

CNS, central nervous system; ECG, electrocardiogram; EEG, electroencephalogram; EHT, engineered human tissues; EMG, electromyogram; JET, jacketed external telemetry; MEA, microelectrode array; UHD, ultra-high definition.

### 3.1 3Rs applied to cardiovascular safety pharmacology assessment

Cardiovascular side effects remain a leading cause of drug attrition (Cook et al., 2014; Weaver and Valentin, 2019). In alignment with the ICH S7A guidance (International Council for Harmonisation (ICH), 2000), the cardiovascular system is one of the three vital organ systems that must be assessed in the safety pharmacology ‘core battery’. Notably, this guidance—published over two decades ago—already acknowledged the relevance of complementary *ex vivo* and *in vitro* models and advocated the use of unanesthetized, unrestrained, and minimally stressed animals for *in vivo* investigations.

The subsequent ICH S7B guideline (International Council for Harmonisation (ICH), 2005b) further strengthened the regulatory framework by mandating both an *in vitro* hERG assay and an *in vivo* QT interval study to evaluate proarrhythmic risk. Even prior to these regulatory milestones, researchers had long employed a variety of *in vitro* and *ex vivo* systems—such as canine or rabbit Purkinje fibers, and isolated cardiac tissues or vessels—to investigate potential cardiovascular effects of drug candidates. The introduction of the Comprehensive *in vitro* Proarrhythmia Assay (CiPA) initiative in 2013 marked a paradigm shift, promoting the integration of *in silico* and *in vitro* methodologies for a more mechanistic and predictive assessment of proarrhythmic potential (Cavero and Holzgrefe, 2014). This initiative catalyzed extensive collaborative efforts aimed at developing, validating, and implementing novel tools, including computational models, ion channel patch clamp assays, and human stem cell-derived cardiomyocytes.

More recently, the rapid evolution of artificial intelligence and machine learning, coupled with significant technological advancements, has further accelerated this transformation, enabling more sophisticated and higher throughput approaches to cardiovascular safety evaluation. While not exhaustive, the following section outlines key *in silico* and *in vitro* assays that are either routinely employed or newly introduced in the field. Additionally, it highlights selected efforts undertaken by the safety pharmacology community over the past two decades to refine *in vivo* study approaches.

#### 3.1.1 From the CiPA paradigm to the revised ICH E14/S7B guidance

Prolongation of the QT interval and the associated risk of Torsade de Pointes (TdP) is recognized as a significant cardiovascular risk in drug development. At the cellular level, the slowing of cardiac repolarization is usually a consequence of inhibition of the rapid component of the delayed rectifier potassium current (I_Kr_). This current is conveyed by the human ether-à-go-go-related gene, also known as hERG, encoding the K_V_11.1 potassium channel (Sanguinetti et al., 1995). Electrophysiological assessment of the hERG ion channel is now routine practice within drug development and uses recombinant cell lines engineered to express a functional human hERG channels. These transfected cell lines have progressively replaced former assays based on animal cells such as Xenopus oocytes, considered less sensitive.

**Figure 2:**
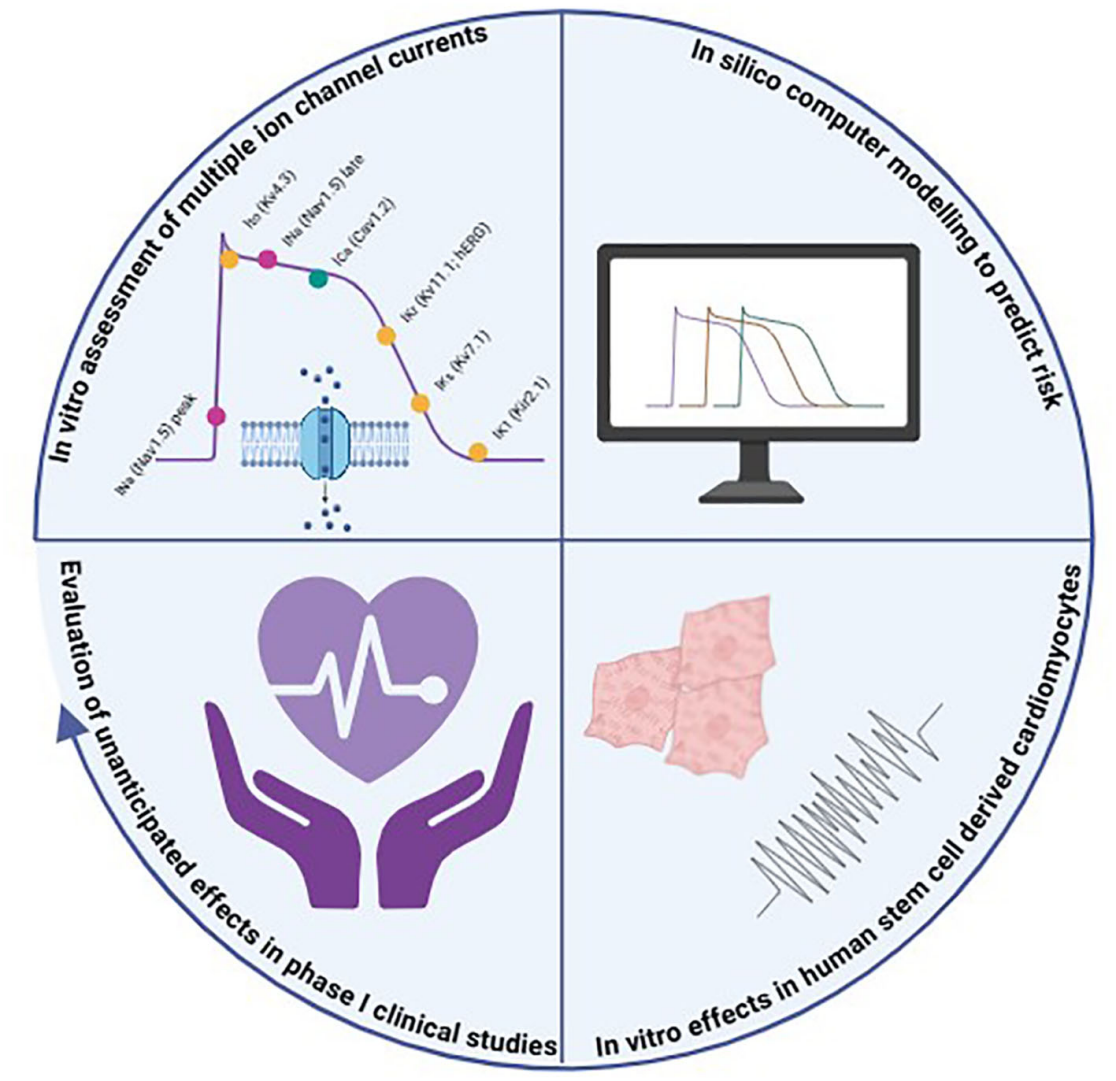
Comprehensive *In Vitro* Proarrhythmia Assessment (CiPA) paradigm consists of three non-clinical pillars complemented by robust ECG monitoring in Phase 1 clinical trials. Created with BioRender.com.

The introduction of hERG screening hugely improved identification of compounds that delay ventricular repolarization to the point that the risk of approving drugs with the potential to induce TdP in patients was effectively eliminated (International Council for Harmonisation (ICH), 2005a, 2005b). However, the high sensitivity of this approach came with low specificity and drugs could be labelled as torsadogenic even if they actually posed no TdP risk. This was attributed to the result of focusing on only one ion channel governing ventricular repolarization (Simpson et al., 2025). Indeed, the cardiac action potential is governed by activity of many ion channels, and cardiotoxicity itself can be caused by other factors (e.g. structural damage). This led to the development of the CiPA initiative which aimed to develop a new paradigm for assessing proarrhythmic risk—utilizing new technologies and an expanded understanding of cardiotoxicity beyond hERG block. CiPA consists of three non-clinical pillars: i) assessment of drug effects on the critical human ventricular ion channel currents; ii) *in silico* integration of the ion channel effects to determine the net effects on the cardiac action potential; iii) a check for discrepancies in fully integrated biological systems (*in vitro* human stem cell-derived cardiomyocyte models) (Sager et al., 2014; Gintant et al., 2016). These non-clinical pillars are complemented by robust electrocardiogram (ECG) monitoring in Phase 1 clinical trials (**Figure 2**).

In 2022, the ICH E14/S7B guidance was revised through a Q&A process and now integrates the 3 CiPA pillars as optional ‘follow-up assays’ (International Council for Harmonisation (ICH), 2022). Despite the fact that CiPA shaped the ICH S7B Q&As, there are no publicly available statistics on real-world regulatory acceptance rates of CiPA assays or published cases where CiPA data were decisive in approving or rejected a drug. Such studies are rather considered as informative to complement core studies and contribute to the integrated non-clinical/clinical risk assessment and allow sponsors to possibly obtain a waiver for the clinical TQT study, under two possible scenarios.

##### 3.1.1.1 Additional ion channel assays

The CiPA ion channel panel contains seven ion current measurements: I_Kr_ (hERG), I_CaL_ (L-type Ca_V_1.2), I_Na_ (Na_V_1.5 peak and late current); I_to_ (K_V_4.3); I_Ks_ (KCNQ1 + KCNE1), and I_K1_ (Kir2.1). These were selected based on their fundamental roles in the cardiac action potential (see **Figure 2**) and documented mutations relating to cardiac arrhythmias by the Ion Channel Working Group (ICWG), sponsored by the Safety Pharmacology Society (SPS) (Colatsky et al., 2016). However, based on a validation study evaluating the effects of 30 clinical drugs, the most critical ion channels were considered to be hERG, Na_V_1.5 late current, and Ca_V_1.2, as blockade of the I_NaL_ and I_CaL_ currents can mitigate I_Kr_ block-mediated delayed repolarization (Crumb et al., 2016). These 3 channels are also the ones highlighted by the ICH S7B Q&A (International Council for Harmonisation (ICH), 2022), for which best practices and detailed protocols have been defined by FDA. As mentioned previously, typically, ion channel assays consist of measuring the ion current by manual or automated whole cell patch clamping of transfected mammalian cells stably expressing the human channel of interest.

##### 3.1.1.2 In silico proarrhythmia risk assessment models

The past decade has seen rapid maturation of *in silico* approaches for predicting cardiotoxicity, with many researchers all over the world developing and validating mechanistic cardiac cell models that can accurately predict drug-induced TdP risk based on ion channel information (Mirams, 2023; Wang and Rodriguez, 2025). These cell models can integrate the effects of the drugs on the various cardiac ion channels and simulate the corresponding changes in the human action potential, offering a more specific prediction than simply measuring hERG inhibition alone.

The input data are typically gathered from *in vitro* screening assays and consist of parameters describing concentration-effect curves such as IC_50_ values for various cardiac ion channels. The output data are one or more biomarkers used for risk predictions. The risk could be represented binary (e.g. a drug is defined as risky or safe), using categories (e.g. low/medium/high risk) or as a continuous measure, with thresholds for risk defined based on known drugs. Many biomarker options have been proposed and published in validation studies, including for example: the net charge carried by the main repolarizing ionic currents over the course of the cardiac action potential or qNet (Li et al., 2019), the occurrence of repolarization abnormalities (Passini et al., 2017), shortening of the electro-mechanical window (Passini et al., 2019), combinations of multiple biomarkers used an input for machine learning-based classifiers (Lancaster and Sobie, 2016; Llopis-Lorente et al., 2020). More recent studies showed how: the use of diseased sub-population, such as hypertrophic cardiomyopathy patients, could improve risk predictions (Christophe, 2022); whole-heart models could provide additional insights into drug-induced arrhythmia mechanisms (Sahli Costabal et al., 2018); cardiac models could be extended to provide predictions of drug-induced changes in contractility and not only electrophysiology (Trovato et al., 2025).

Overall, human *in silico* drug trials are a powerful method for preclinical drug assessment allowing for high throughput concurrent electrophysiological and inotropic drug assessment. A critical factor for the success of these models has been incorporating population variability (Muszkiewicz et al., 2016) and uncertainty quantification (Chang et al., 2017), both essential elements when aiming at predicting risk over the whole population. From a 3Rs perspective, these *in silico* methodologies offer accessible opportunities to replace *ex vivo* animal models, such as rabbit Purkinje fibers (Mohr et al., 2022; Trovato et al., 2022) or Langendorff isolated rabbit heart (Holmes et al., 2025) in the near future. The CiPA initiative has been one of the main drivers behind a wider inclusion of *in silico* cardiac models for cardiac safety assessment in the drug development pipeline, and the general principles to consider for the validation of proarrhythmia risk prediction models—published as a white paper in 2019 (Li et al., 2020)—constitute a good starting point towards the harmonization of approaches.

##### 3.1.1.3 From ex vivo animal tissues to *in vitro* human cardiac organoids

**Figure 3:**
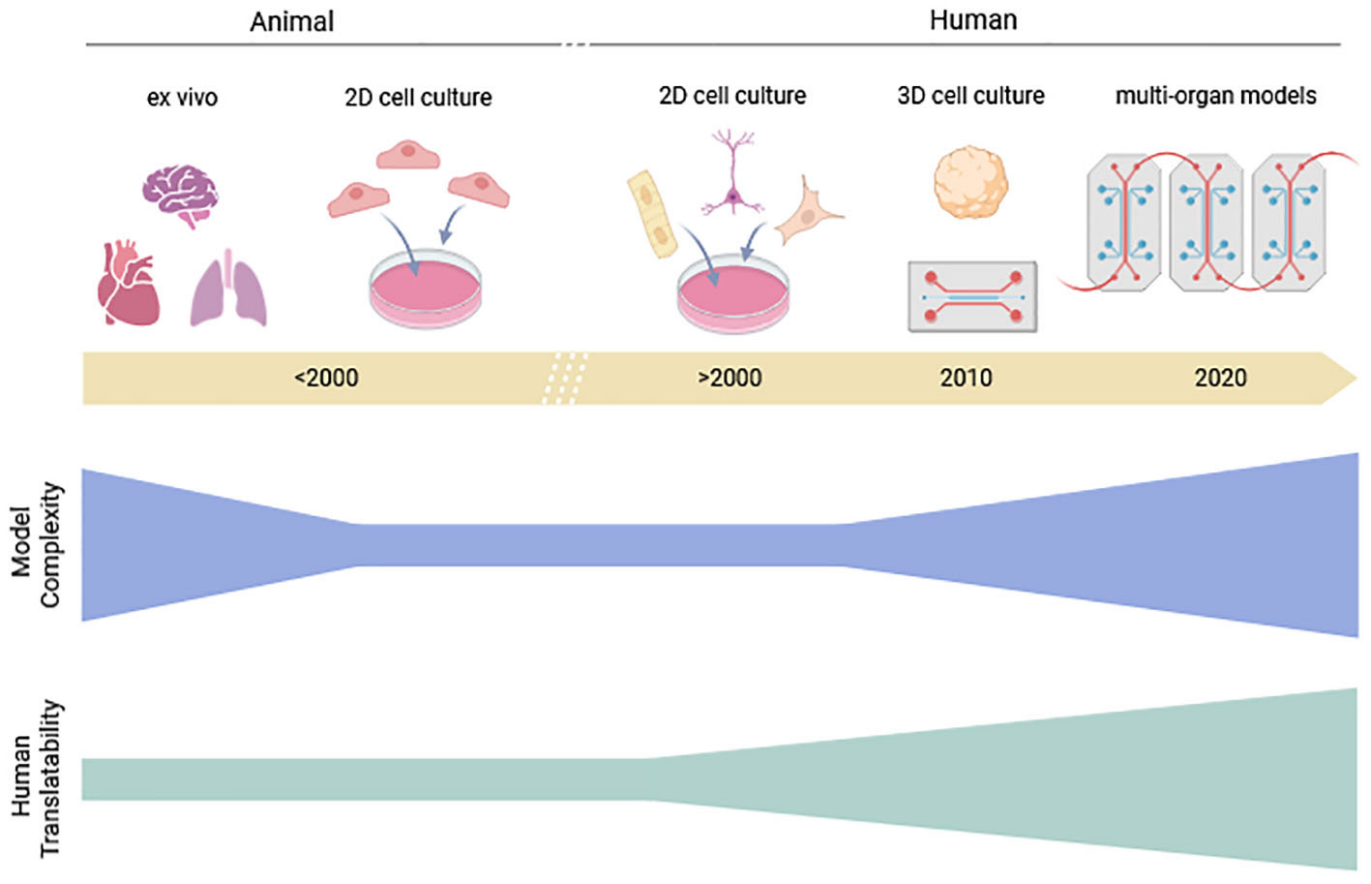
Relationship between model complexity and human translatability of *ex vivo* and *in vitro* methods used in preclinical development. The lowest complexity is observed with 2D cultures derived from animal or human sources. Human translatability represents the extent to which a model reflects human physiological conditions and is generally regarded to be higher in models based on human-derived material due to species equivalence. Created with BioRender.com.

**Figure 3** illustrates the progressive evolution of *in vitro/ex vivo* methods used in preclinical development and the relationship between model complexity and human translatability, i.e. the extent to which a model reflects human physiological conditions.

Since its introduction in 1895, the isolated perfused heart technique has been a key tool for studying cardiac function *ex vivo* (Langendorff, 1895). A vertebrate heart, typically from a rodent (e.g. rat and guinea pig) or a non-rodent (e.g. rabbit), is excised and retrogradely perfused with oxygenated solution in a temperature-controlled bath, enabling short-term viability for assessing mechanical and electrical responses to drugs. The setup is usually used for early cardiac safety screening measuring left ventricular pressure, coronary flow, action potential, and electrophysiological activity, for evaluating the direct chronotropic, inotropic, and proarrhythmic effects on the heart. However, limitations include animal sacrifice, raising ethical concerns amid efforts to reduce testing (e.g., FDA Modernization Act 2.0), and physiological differences between rodent/non-rodent and human hearts including heart rate, ion channel distribution, and metabolic profiles that hinder translation. The absence of neurohumoral and immune interactions further restricts applicability to whole-organism studies.

Myocardial tissue slices have emerged as a refined alternative to whole-organ models like the Langendorff heart. Ultrathin sections (100–400 µm) are prepared via vibratome slicing and maintained in assay buffer, preserving viability for hours. Retaining native architecture and cell-cell junctions, slices bridge isolated cell cultures and intact organs. However, reliance on animal tissue and low throughput limit broader use in safety pharmacology. Similarly, the Purkinje fiber assay is a well-established *in vitro* method for assessing proarrhythmic risk. Purkinje fibers, typically from rabbit hearts, are superfused with a physiological solution and paced electrically. Intracellular microelectrodes record action potentials, enabling precise measurement of action potential duration, resting potential, and early-(EADs) and delayed-after depolarizations (DADs).

Since the early 2010s, human induced pluripotent stem cell-derived cardiomyocytes (hiPSC-CMs) represent a major advancement in preclinical cardiac research, aligning ethical and scientific priorities by enabling the exclusive use of human biological material (**Figure 3**). hiPSC-CMs have emerged as a valuable tool in early-stage drug screening and mechanistic studies due to their ability to mimic key aspects of human cardiac physiology. Two-dimensional (2D) models offer the potential to fully replace animal experiments and improve translational relevance. Although they exhibit fetal-like electrophysiological and structural characteristics, including incomplete ion channel expression, calcium handling, and metabolic development (Yang et al., 2022; Lu et al., 2025), ongoing maturation strategies are steadily improving their functional fidelity. For example, prolonged culture improves sarcomeric organization and electrophysiology, though incompletely (Yang et al., 2023) while electrical pacing and mechanical stimulation enhance action potentials, calcium handling, and contractile force (Lu et al., 2025). Biochemical cues such as thyroid hormone, dexamethasone, and fatty acids promote metabolic maturation (Malan et al., 2025). Three-dimensional approaches and engineered tissues provide more physiological environments (Ruan et al., 2016), while co-culture with fibroblasts and endothelial cells supports structural and functional development (Giacomelli et al., 2020). Because no single method fully mimics adult cardiomyocytes, combined mechanical, electrical, biochemical, and architectural cues are increasingly essential.

Among the assays used to assess cardiac safety profiles using hiPSC-CMs, microelectrode array (MEA) technology, introduced in the early 2000s, has become central, enabling non-invasive, multi-site recordings of extracellular field potentials and detection of proarrhythmic risk (Meyer et al., 2004). MEAs overcome limitations of hERG assays and animal models by capturing complex repolarization dynamics with higher throughput (Pradhapan et al., 2013) and by providing human-like electrophysiological profiles with improved accuracy, in alignment with the CiPA initiative (de Korte, 2017). However, to improve confidence in such NAMs, it is crucial to understand the reproducibility and consistency across platforms and laboratories. The Japan iPS Cardiac Safety Assessment (JiCSA) initiative has been established under national government policy to develop and validate hiPSC-CM assays for proarrhythmia risk assessment. To establish standardized protocols and assess reproducibility across laboratories, JiCSA first performed validation study of hiPSC-CM-based assays in collaboration with academia, industry, and regulatory stakeholders, providing the initial scientific multisite study using (Nakamura et al., 2014). Following the principles outlined in the OECD Guidance Document 34 (GD34) for validation and regulatory acceptance of new test methods, JiCSA designed a comprehensive strategy to evaluate reproducibility, reliability, and clinical relevance (Kanda et al., 2016). By assessing parameters, such as cell density, culture conditions, plate coating, and detection of EAD-like events, JiCSA conducted a large-scale, multisite validation study involving sixty compounds with different torsadogenic risks, including CiPA 28 compounds. The results demonstrated high predictivity when compared with a public database called CredibleMeds (Ando et al., 2017). A notable finding was that using unbound (free) drug concentrations, rather than total concentrations, significantly improved predictive accuracy. JiCSA further confirmed this refinement by re-analyzing the international CiPA dataset, demonstrating that predictive performance also improved when free drug concentrations were considered (Kanda et al., 2018). Both initiatives produced largely consistent results, and JiCSA’s emphasis on unbound drug concentrations offered a valuable refinement to the global best practice discussion on proarrhythmia risk assessment.

Today, MEAs are widely used for detecting drug-induced arrhythmias and electrophysiological effects, alongside optical tools such as calcium imaging and voltage-sensitive dyes (VSDs) which enable high-resolution mapping of electrical (Lee et al., 2012) and calcium dynamics (Herron et al., 2012). Genetically encoded indicators (GECIs, GEVIs) provide cell-type specificity and long-term imaging, though their regulatory use is still emerging (Mollinedo-Gajate et al., 2019).

Engineered Heart Tissues (EHTs) (also called cardiac organoids) represent a significant progress in the field, offering three-dimensional (3D) constructs that recapitulate key aspects of native myocardial architecture and function (Mannhardt et al., 2016) and enable the assessment of contractile force and electrophysiology in response to pharmacological stimuli in more physiologically relevant environments than conventional 2D monolayer cultures (**Figure 3**). Complementary higher-throughput 2D platforms like the FLEXcyte 96 offer scalable, cost-efficient measurement of contractility and beat dynamics (Goßmann et al., 2016, 2020) (Lickiss et al., 2022, 2024), supporting early-stage cardiac safety screening within the NAMs framework. Despite the lack of pharmacokinetic and multi-organ integration contexts, the expanding role of these hiPSC-CM models reflects both scientific progress and alignment with ethical imperatives to reduce animal testing (Begovic et al., 2024).

Beyond acute functional readouts such as electrophysiological or contractility changes, the integration of structural biomarkers of cardiotoxicity is increasingly recognized as essential for optimizing the predictive value of the hiPSC-CM models. Predicting morphological damage to cardiac cells or their substructures after chronic drug exposure—particularly with agents such as anticancer drugs—remains a significant challenge in preclinical safety assessment. **Table 4** summarizes the main types of structural biomarkers and their detection methods currently used in hiPSC-CM assays.

**Table 4:** Functional and structural biomarkers of cardiotoxicity in human stem cell-derived cardiomyocyte assays.

Biomarker type	Endpoints	Detection method	Key references
Electrophysiological changes	APD_90_, FPD, beat rate, sodium spike amplitude	Voltage sensitive dye, MEA, calcium flux	(Blinova et al., 2017)
Contractility/beat rate	Amplitude, beat rate, contraction slope, relaxation slope, contraction duration, relaxation duration, area under the curve	Contraction/force measurement, imaging, impedance	(Goßmann et al., 2020; Lickiss et al., 2022)
Viability/cytotoxicity	ATP, LDH, Cell titer	Luminescence, fluorescence	(Talbert et al., 2015; Bassett et al., 2025)
Protein injury markers	Troponin I (cTnI), troponin T (cTNT), NT-proBNP, FABP3	ELISA, immunoassay	(Talbert et al., 2015; Kopljar et al., 2017; Adamcova et al., 2019)
Metabolites (‘metabolomics’)	Arachidonic acid, lactic acid, thymidine, 2’-deoxycytidine, creatine, glutathione	LC-MS, UPLC-HRMS	(Palmer et al., 2020; Bowen et al., 2025)
Micro-RNAs	miR-133b, miR-184,miR-208b-3p, miR-182-5p	RT-qPCR, sequencing	(Gryshkova et al., 2022; Cherubin et al., 2025)
Morphological features	Cell/organelle morphology, sarcomere integrity	High content imaging	(Homan et al., 2021; Rosell-Hidalgo et al., 2024; Szabo et al., 2025)

APD_90:_action potential duration at 90% repolarization; ATP, adenosine triphosphate; cTnI, cardiac troponin I; cTnT, cardiac troponin T; FABP3, fatty acid binding protein 3; FPD, field potential duration; HRMS, high resolution mass spectrometry; LC-MS, liquid chromatography-mass spectrometry; LDH, lactate dehydrogenase; MEA, microelectrode array; NT-proBNP, N-terminal pro-brain natriuretic peptide; RT-qPCR, reverse transcription quantitative polymerase chain reaction; UPLC, ultra performance liquid chromatography.

A multiplexed approach, combining several biomarker classes, provides a comprehensive screening tool that enhances early detection of cardiac toxicity. Among the most validated markers, changes in specific extracellular metabolites—such as arachidonic acid, lactic acid, thymidine, and 2′-deoxycytidine—are highly predictive of both functional and structural cardiotoxicity. These metabolites are measured in the culture media following compound exposure, and their levels, often combined into biomarker ratios, have been shown to correlate with clinical outcomes (Palmer et al., 2020).

Recent research has also highlighted the value of specific microRNAs (miRNAs) as sensitive and translatable biomarkers of structural cardiotoxicity. Key miRNAs identified include miR-133b, miR-184, miR-208b-3p, miR-187-3p, and miR-182-5p (Gryshkova et al., 2022). Their upregulation in hiPSC-CMs (intracellular) or in the culture media (secreted) after drug exposure is associated with cardiac injury and provides a translational link to circulating miRNAs measurable in patients. These miRNAs are increasingly recognized as promising endpoints for *in vitro* cardiac safety assessment.

Protein biomarkers, particularly cardiac troponins (cTnI and cTnT), are well-established indicators of cardiomyocyte injury. Their release into the culture medium following structural damage has been validated in hiPSC-CM models and is analogous to their clinical use for detecting myocardial injury (Kopljar et al., 2017).

High-content imaging enables quantification of structural changes at the cellular and subcellular levels, including alterations in cell size and shape, mitochondrial integrity, sarcomere organization, and organelle-specific features such as lysosomes and the endoplasmic reticulum (Rosell-Hidalgo et al., 2024). Morphological profiling, especially when combined with transcriptomic analysis, can outperform other endpoints in predicting clinical structural cardiotoxicity.

Finally, less complex but easily monitorable endpoints such as ATP content or LDH release, while not specific to structural cardiotoxicity, remain essential for distinguishing cytotoxic from sub-cytotoxic effects (Bassett et al., 2025).

The hiPSC-CMs are one of the most mature *in vitro* NAMs submitted to FDA. In their recent analysis, FDA identified 34 hiPSC-CM studies submitted between 2012 to 2020, 65 studies between 2020 and 2023, and 40 studies between 2024 and 2025, representing only a small percentage (approximately 1%) of new commercial IND submissions per year (Simpson et al., 2025). Most of these hiPSC-CMs studies were used in candidate selection phase rather than in early discovery and were submitted alongside traditional hERG and animal QT data. In addition, FDA has performed a predictivity analysis with 31 of those INDs selected between 2020 and 2023, where MEA or voltage-sensitive optical (VSO) platforms were used. Despite several limitations such as small sample sizes and imperfect data quality, hiPSC-CMs were shown to be as predictive as the animal (non-rodent) *in vivo* QT prolongation studies with a concordance value of 0.71, sensitivity of 0.50, and specificity of 0.77. Furthermore, hiPSC-CM and clinical QTc prolongation showed a concordance value of 0.83 with a sensitivity of 0.80 and a specificity of 0.83 (Simpson and Feaster et al., manuscript in review). hiPSC-CMs could replace animals in certain use cases, but a larger dataset, additional resources through external collaborations and increased submission of NAM studies to FDA may help build more confidence in the comparable predictive performance for broader replacement of non-rodents in assessment of proarrhythmia and other cardiovascular endpoints (e.g., cardiac contractility).

In summary, the integration of multiplexed structural biomarkers—including metabolites, miRNAs, protein injury markers like troponin, morphological profiling, and viability assays—provides a robust and translational approach for early detection of drug-induced structural cardiotoxicity in hiPSC-CM models. Their application to 3D cardiac organoids or MPS is an ongoing effort.

#### 3.1.2 Refinement of *in vivo* cardiovascular models

**Figure 4:**
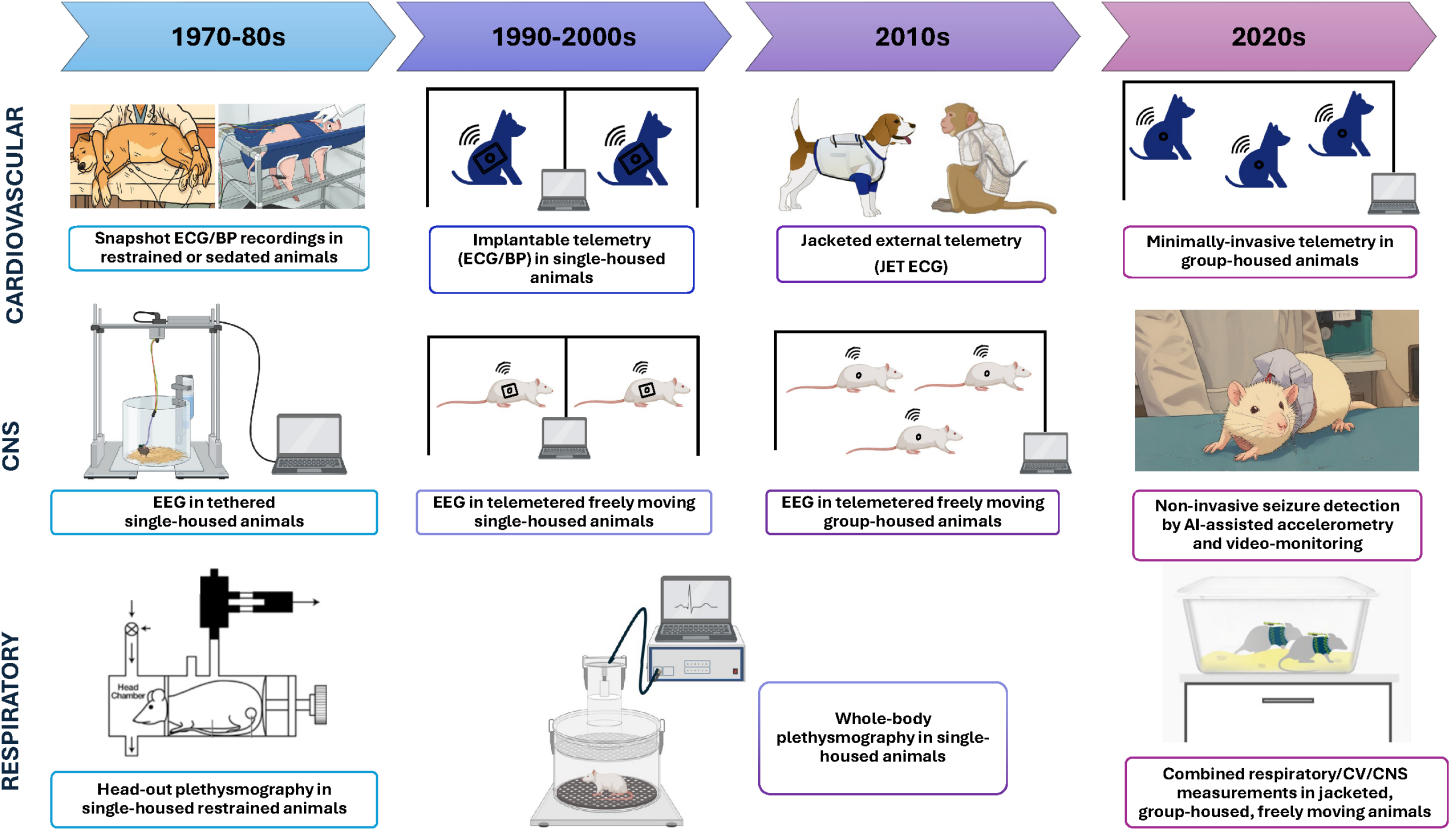
Progressive refinement of the *in vivo* methods used in safety pharmacology core battery studies. Created partly with Canva.com.

A cardiovascular *in vivo* safety pharmacology evaluation in a relevant animal species is an essential component of the non-clinical safety assessment of new drug candidates and is still a mandatory core assay of the ICH S7B guidance. Although the primary focus of such study is on QTc prolongation, it also evaluates drug-induced functional effects on other ECG parameters (PR interval, QRS duration), as well as on hemodynamics (arterial blood pressure (BP), and heart rate), thereby fulfilling the ICH S7A requirements. **Figure 4** shows the refinement over time of the *in vivo* methodologies used in safety pharmacology studies across the three vital organ systems. Before the advent of implantable telemetry devices for laboratory animals in the 1990’s, most cardiovascular studies relied on anesthetized, physically-restrained, acutely instrumented animals or employed invasive and stressful techniques in conscious animals, such as tethered systems. While anesthesia eliminates pain and suffering, all anesthetic agents can significantly alter cardiovascular parameters, potentially confounding the pharmacodynamic response to the test drug itself (e.g., alpha-chloralose-induced tachycardia and increase in pulmonary pressures, isoflurane-induced vasodilation, ketamine-induced tachycardia and hypertension, or barbiturates-induced cardio-depressant effects) (Nagayama et al., 2024). Similarly, stress associated with handling or physical restraint is known to activate the sympathetic nervous system, leading to elevated, non-physiological BP and heart rate values (Balcombe et al., 2004; Crestani, 2016). Moreover, restraint-based methods generally exhibit lower sensitivity and reproducibility compared to telemetry in freely moving animals (Henderson et al., 2025; Valentin et al., 2025a).

Over the years, substantial progress has been made in the miniaturization and refinement of telemetry devices. Today, cardiovascular telemetry can be performed even in small species such as mice and allows maintaining groups or at least pair-housing during data acquisition, aligning with animal welfare legislation and best practices (Skinner et al., 2019; Prior and Holbrook, 2021) (**Figure 4**). Providers have developed dual pressure telemetry implants that allow simultaneously measurement of high quality left ventricular pressure (LVP) and arterial BP from the same animal. This can be used in various species (rodents and non-rodents) for routine safety pharmacology (small molecules) and issue resolution (small and large molecules) studies. This technology allows to optimize animal use, reduce colony size, labor, housing and cost, and reduce compound needs. It is the best alternative to performing two separate telemetry studies (LVP & BP) for myocardial contractility and blood pressure assessment. The revised ICH E14/S7B Questions & Answers (Q&As) regulatory document (International Council for Harmonisation (ICH), 2022) considers the conduct of cardiovascular studies in telemetered non-rodents as the gold standard method for integrated QTc prolongation risk assessment supporting clinical TQT waiver requests.

Recent publications from industry and CRO consortiums (Rossman et al., 2023; Vargas et al., 2023) highlight the importance of defining optimal study practices to enhance the reliability of these studies and improve overall study sensitivity. Such good practices concerning the design, the execution and the analysis/reporting of the study, together with a translational approach (such as concentration-QT modeling), may improve confidence in nonclinical data for regulatory decision-making regarding the proarrhythmic liability assessment of new drugs. Best practices lead to better data, with less animals, and increasing their value for clinical QTc risk assessment.

The classical cross-over design applied in stand-alone cardiovascular telemetry studies exemplifies the application of the Reduction and Refinement principles in safety pharmacology. Indeed, instead of using a parallel dose design with typically 16 animals (e.g., 4 groups of n=4 animals: control and 3 dose levels), a Latin square design requires only 4 animals to achieve comparable or even better scientific outcomes. Taking into account that around 360 small molecule drugs were approved by FDA between 2010 and 2024, applying the cross-over design in the cardiovascular safety pharmacology study has saved more than 4000 non-rodents. This approach not only reduces the number of animals needed but also enhances statistical power and reproducibility, as each animal serves as its own control. Finally, animals can be reused across several studies, provided that an appropriate washout period is applied between treatments, no adverse effects have occurred, and all cardiovascular parameters have returned to baseline. A study performed with 6 reference drugs previously evaluated in a prospective clinical QT study (Darpo et al., 2015) demonstrated sufficient sensitivity with only 4 telemetered dogs in detecting minimal QTc changes (<10 msec), at concentrations close to human clinical (therapeutic) exposure (Darpo et al., 2015; Bétat et al., 2024).

In addition to the stand-alone safety pharmacology study, which evaluates the acute effects of a drug on the cardiovascular system, it is also useful to assess its impact after repeat administration. Indeed, drug-induced cardiovascular effects could be aggravated, remain similar, or disappear after chronic administration. For this purpose, ECGs are often collected during repeat-dose toxicity studies from chemically or physically-restrained animals over a short timeframe. However, manual restraint and the use of anesthesia can induce cardiovascular changes that confound interpretation of ECG measurements, and capturing only a few cardiac cycles provides a limited and potentially non-representative sample of cardiac function. These limitations can be mitigated by using a non-invasive jacket-based ECG system. Jacketed External Telemetry (JET) enables continuous measurement of ECG and heart rate without requiring surgery, reducing stress-related artefacts and improving data quality. It nevertheless needs an acclimation period for the animals, which is critical for the JET data quality and sensitivity to detect drug-induced changes in cardiac conduction and repolarization (Derakhchan et al., 2011a). JET-derived PR, QT, QTc intervals, QRS duration, and heart rate correlated well with those derived from PCT telemetry transmitter (Derakhchan et al., 2014). JET ECG is a great example for Refinement and a preferred method compared to restraint-based ECG because high-density ECG sampling can be collected in unstressed conscious large animals, in short- and long-term studies of 1–7 months duration (Vargas et al., 2010; Chui et al., 2011; Derakhchan et al., 2011b, 2011a). The JET BP add-on acquires accurate and continuous BP data from a minimally invasive implant, and JET RIP (Respiratory Inductive Plethysmography) can continuously monitor respiratory rate and depth with the jacket abdominal and rib cage bands.

In certain circumstances, it might be required to assess additional parameters outlined in the follow-up studies section of the ICH S7A guidance—such as cardiac output, myocardial contractility, and vascular resistance—for a deeper understanding and mechanistic investigation of drug-induced changes in these cardiovascular parameters. This may, therefore, necessitate additional studies. However, recent progress has focused on extracting these additional parameters directly from the cardiovascular telemetry study itself. This advancement—supported by *in silico* modeling—has the potential to replace some follow-up investigations, thereby further aligning with the 3R principles by both reducing the need for supplementary studies and refining the existing evaluation through the integration of advanced derived cardiovascular endpoints (Champeroux et al., 2024). Pulse contour analysis of arterial waveforms enables estimation of stroke volume, cardiac output, and systemic vascular resistance. Central aortic pressure can be approximated using the N-point moving average (NPMA) method for beat-to-beat modeling, improving accuracy and cross-species applicability (Champeroux et al., 2024). Generalized transfer functions have been developed for dogs to enhance waveform reconstruction (Hotek et al., 2023). Modeling pulsatile arterial hemodynamics and ventricular-arterial coupling has advanced further. Analyses using BP and synthesized aortic flow evaluate pulse wave propagation, wave reflection, arterial impedance, cardiac efficiency, and wave power. Synthesized flow waveforms have shown high concordance with measured flow, providing mechanistic insights into cardiovascular dynamics and drug effects. While promising, routine implementation remains challenging, and integration with AI/ML could enhance usability (Hotek et al., 2023). High-Frequency Autonomic Modulation (HFAM) evaluates autonomic control of heart rate through high-frequency oscillations. The HFAM index differentiates parasympathetic dominance (S1), co-activation of sympathetic and parasympathetic systems (S2), and sympathetic predominance (S3). This approach detects compensatory autonomic responses that may be missed by standard BP analyses (Champéroux et al., 2018, 2022).

Overall, refinements in telemetry technologies, combined with *in silico* modeling, advanced hemodynamic analyses, and HFAM, expand cardiovascular endpoints, enhance mechanistic insights, and reduce animal use. While full replacement of *in vivo* models is currently limited, these approaches improve the translational value of preclinical studies to the clinical setting.

### 3.2 3Rs applied to CNS safety pharmacology assessment

Retrospective analyses and industry surveys demonstrate that many common CNS liabilities are slipping through the current screening paradigm, becoming evident in Phase I clinical trials (Cook et al., 2014; Authier et al., 2016; Morgan et al., 2018; Authier et al., 2025; Jackson et al., 2019). Therefore, there is a clear need for earlier and more predictive CNS liability detection with reduced reliance on animal studies. Ideally, *in vitro* models should accurately recapitulate human brain physiology and have potential for a high-throughput approach amenable to compound screening. Given the varied nature and severity of CNS toxicities which span from headache or fatigue to gait abnormalities, hallucinations, or seizures, it is challenging to develop *in vitro* methodologies able to detect all these outcomes and recapitulate the complexity of the human brain anatomy and function. Interestingly, unlike the cardiovascular safety field where the CiPA initiative stimulated substantial efforts to develop and validate predictive tools, ultimately leading to their inclusion in the revised ICH S7B guidance in 2022, no comparable initiative has emerged for CNS safety. The former European IMI2-NeuroDeRisk project (2019-2022) aimed to enhance the predictivity of preclinical models for detecting neurotoxicity, with particular emphasis on approaches that are in line with the 3Rs principles. Additionally, a HESI working group sought to identify translational biomarkers of neurotoxicity and neurodegeneration (Walker et al., 2018). However, these collaborative efforts have not yet resulted in any formal regulatory guidance and at the time being, CNS NAMs are not ready to fully replace *in vivo* studies. Nevertheless, the field is rapidly progressing with increasing interest in *in silico* and *in vitro* tools, in particular for seizure prediction and blood brain barrier simulation.

#### 3.2.1 From the historical Irwin test to smart cages

In contrast with the cardiovascular safety assessment of pharmaceuticals which is regulated through two dedicated guidelines, there is no or little regulatory framework on CNS non-clinical safety assessment, except for drug abuse liability. Although CNS is one of the three vital systems that form part of the core battery of safety pharmacology studies, the ICH S7A guidance provides sparse information to drug developers with regard to models or studies to focus on. Typically, the most common approach to assess potential CNS side effects is the historical Irwin test (Irwin, 1968), adapted as ‘functional observational battery’ (FOB) for safety assessment purposes (Baird et al., 1997; Redfern et al., 2005; Gauvin and Baird, 2008; Himmel, 2008). This neurobehavioral test is usually conducted in rodents but has also been adapted to larger species, and aims to identify a wide range of autonomic, neuromuscular, sensorimotor, and behavioral effects after administration of a test compound.

The Irwin/FOB relies on highly experienced operators and should be performed blinded to ensure that all endpoints are detected in an objective manner. However, it is still possible that some CNS-related signs may be overlooked, especially if they are sporadic and subtle. The assessment is generally conducted in a sequential order, with the animal first observed in its home cage, then placed in a new environment to assess its exploratory behavior and finally handled by the operator to evaluate some reflexes and record some parameters such as body temperature. These observations can be repeated at different timepoints after drug administration. Despite its inherent subjectivity and interlaboratory variability, and its poor predictive value for some side effects such as headache, dizziness or somnolence/fatigue (Mead et al., 2016), the FOB remains an interesting safety pharmacology tool to characterize the neurobehavioral profile of a lead compound or drug candidate, in particular at early stage of a project (Redfern et al., 2019).

The recent progress in AI/ML has triggered various initiatives to refine neurobehavioral assessments by recording behavioral and motor activity parameters directly from the home cage in a continuous manner (24/7). Data are analyzed by various algorithms able to detect different behaviors (supported rearing, grooming, tremors, stereotypies, sleeping, walking, …) (Chen et al., 2023; Chindemi et al., 2025). This avoids the repeated stress of the animals due to handling and allows to detect potential CNS-related signs occurring during the night. A great example of these ‘smart cages’ is the newly developed automated VivaMARS activity system rack, with each rack having 10 sensor boxes, and each sensor box consists of over 180 infrared beams, for up to 30 animals per session. These systems can be used in the setting of general toxicology studies, or eventually in pharmacology studies, avoiding the need to conduct stand-alone Irwin studies, thereby contributing to the Reduction and Refinement principles (Beck et al., 2025). The only challenges are the associated cost of such sophisticated equipment and the huge amount of generated data that need to be recorded, stored, and analyzed.

On the top of the classical, standard Irwin/FOB that is part of the core battery of safety pharmacology studies and already covers a wide range of potential CNS side effects, some pharmaceutical companies may decide to develop their own CNS safety derisking strategy to focus on side effects that could constitute a stopper in a project such as seizures, or for a particular patient population (e.g., hypertension in diabetic patients, hypotension in Parkinsonian patients,…).

#### 3.2.2 3Rs for seizure liability assessment

Seizure is a particularly serious side effect defined as uncontrolled electrical activity in the brain. Seizures may not necessarily lead to ‘clinical’ convulsion (Pitkänen et al., 2006) and can only be detected using electroencephalogram (EEG) recordings, meaning they will be missed using the workflow that is reliant on observed behavioral alterations. This allows a significant loophole as approximately 75% of clinical compounds with seizure did not show convulsions in nonclinical safety studies in a retrospective Japanese study (Nagayama, 2015). As proposed by Easter et al. (2009) (Easter et al., 2009), the use of a stepwise cascade of assays is a useful approach to derisk seizure liability from early stages of discovery projects. This enables the early elimination of seizurogenic compounds based on *in silico* and/or *in vitro* results, thereby avoiding animal testing and preventing unnecessary harm.

##### 3.2.2.1 *In vitro* seizure derisking assays

Early seizure liability assessment typically begins with *in vitro* pharmacological profiling, to identify interactions with seizure-associated targets. However, there is no industry consensus on a standard ‘seizure’ panel. Easter et al. (2009) identified 53 targets, with 26 included in standard secondary pharmacology panels (Bowes et al., 2012; Brennan et al., 2024). Of Brennan’s 33 proposed novel targets, 14 relate to CNS adverse effects. Recent work integrating adverse outcome pathways (AOPs), NAMs, and *in silico* approaches highlights the need for expanded panels, focusing on neurotransmitter receptors, transporters, enzymes, and voltage- and ligand-gated ion channels (Behl et al., 2025). Mutations in some neuronal ion channels (e.g., Na_V_1.1, K_V_7.2, GABAα1β2γ2, NMDA) disrupt electrical signaling and predispose to seizures; compounds mimicking these effects may cause adverse events. Similar to CiPA for cardiac safety, ion channel screening could improve seizure risk prediction. Automated patch-clamp assays for seizure-related channels are offered by CROs, and recent studies (Rockley et al., 2023; Cohen et al., 2025) support Ca_V_2.1 screening as predictive. Functional assays outperform binding assays, as shown by a case study where GABA_A_ inhibition was missed by binding screens (Delaunois et al., 2020). A condensed ion channel panel representing key families could serve as a broad CNS safety screen, given overlap between seizure, cognition, and mood endpoints.

In addition to the *in vitro* off-target profiling, *in vitro* cellular or tissular assays assessing changes in the normal function of a neuronal network can provide valuable insights to understand the seizurogenic potential of new molecules, in particular if they affect several ion channels or targets in parallel.

Although rodent hippocampal brain slices continued to be used by approximately 40% respondents of a nervous system focused industry survey (Authier et al., 2025), the use of stem cell-derived neurons has gained huge traction, since it increased from 14% in 2016 to 50% in 2025, while co-cultures of neuronal and glial cells raised from 17% to 44% during the same period. The MEA technology is the most common method to measure the electrical signaling of networks of neuronal cells *in vitro*. Heterogeneous mixtures of neuronal cells (i.e. glutamatergic neurons, GABAergic neurons, astrocytes) are seeded on specialized plates and cultured until they have formed synaptic connections and fire synchronously. Once the cells are firing synchronously, changes to the firing pattern are recorded after adding compounds. It is possible to identify compounds from different classes using this technique as they can give different fingerprints of seizurogenic activity (Bradley et al., 2018). This technique has shown promising results in several studies for detection of seizurogenicity and has been conducted using both primary rodent cells and human derived induced pluripotent stem cells (hiPSCs). The study by Bradley et al. using primary rodent cortical neurons accurately predicted the seizurogenic potential of 14 out of 15 known proconvulsant compounds and classified 2 clear and distinct patterns of seizurogenic responses based on compound class (Bradley et al., 2018). Similar studies also demonstrate accurate identification of known convulsants and suggest that the *in vitro* MEA assay with rat primary neurons may have advantages over an *in vivo* rat model (Tukker et al., 2020; Kreir et al., 2018). Human iPSCs representative of key cellular subtypes present in the brain have also been utilized on the MEA with promising results for identification of seizurogenicity. The IMI2-NeuroDeRisk project compared primary rat cortical neurons and hiPSC-derived neurons co-cultured with hiPSC-derived astrocytes on two different MEA systems across three separate laboratories. Activity of 33 compounds categorized as positive tool drugs, seizure-positive or seizure- negative compounds was evaluated. Overall, the study found that hiPSC-derived neurons are equivalent or even more sensitive than rat cortical neurons in detecting drug-induced seizurogenic activity; therefore, both methods are of use within early drug discovery (Zhai et al., 2023). There are several limitations pertaining to the use of hiPSCs—namely the artificial nature of the network which may not fully recapitulate complex *in vivo* and human networks. Primary rodent cortical cells have the benefit that all neuronal cell types will be present in the preparation, however from a 3R perspective, the use of hiPSCs for early screening is preferable, and efforts to standardize the cell maturation and validate the assay with a broad range of pharmaceutical agents continue.

##### 3.2.2.2 Refinement of *in vivo* seizure models

EEG monitoring is the gold standard approach for seizure detection. In safety pharmacology, EEGs are generally not conducted routinely, but only on a case-by-case basis, to confirm that convulsions eventually observed in an *in vivo* study are of CNS origin, or to better characterize or understand the mechanisms behind this clinical observation. The use of telemetry was first introduced several decades ago as a refinement since it allows group-housing of freely moving animals (**Figure 4**), but still requires surgery for permanent implantation of brain electrodes, for up to 150 days of continuous EEG recording in rodents and non-rodents. Moreover, it is a quite expensive technology with limited throughput requiring 21 days of post-surgery recovery, but it has been used by 67% of ACT-SPS-STP 2024 CNS Industry survey participants (Authier et al., 2025). Non-invasive methods have been recently developed for laboratory rodents, based on ultra-high-definition video-monitoring assisted by machine learning (Diaz-Arce et al., 2022; Sabnis et al., 2025). Accelerometry, which combines video-monitoring and movement detection, is also an interesting alternative approach to tethered or telemetered EEGs. Al-algorithms are able to detect convulsive/epileptic events, which need thereafter to be confirmed/validated manually by a human operator, as the number of false positives may be relatively high. Although primarily developed for epilepsy research, these techniques are also applicable to safety assessment for detection of drug-induced seizures. Another example of refinement is the non-invasive jacketed EEG with temporary electrodes placed under a jacket to record EEG in dogs for 24–48 hours and up to four bipolar EEG derivations.

#### 3.2.3 Novel approaches for other CNS liabilities

For some CNS liabilities such as suicidality or drug abuse potential, it is generally considered that there is little or no alternative to *in vivo* models. Suicide or depression is a complex behavior which is impossible to fully reproduce in animals. Some behavioral endpoints in some animal species have however been proposed as predictive markers of depression, such as anhedonia, aggressivity, and helplessness in rodents, or lack of peer-rearing in primates. They are considered as refined approaches compared to historical ‘despair’ tests like the forced swim or tail suspension tests, which are highly controversial due to the perceived severity for the animals (Sewell et al., 2021; Mohseni and Rafaiee, 2025). However, these behavioral traits are still associated with significant challenges and drawbacks (Planchez et al., 2019). Some novel approaches have emerged such as proteomics or micro-RNAs but remain anecdotical at this stage (Wang and Dwivedi, 2025).

From a regulatory perspective, abuse liability studies are expected for any new CNS-active drug and are generally conducted before starting Phase 3 clinical trials. Abuse liability studies in animals are very heavy, expensive, ethically questionable, and time-consuming. However, both EMA and FDA agencies infer that the decision on abuse liability assessment is dependent on the level of risk or concern that is determined case-by-case for each drug. Therefore, it is possible to waive the animal studies (Derakhchan et al., 2023), after evaluation of the dossier by the regulatory agencies and based on the results of the ‘eight-factor analysis’. This includes for example *in silico* assessment (chemical structure similarity with known drugs of abuse), *in vitro* pharmacology profiling data (affinity/activity on targets involved in abuse liability), PK data (brain penetration, rapid absorption, fast onset), Irwin/FOB data (e.g., head twitches in mice), general toxicology data (withdrawal signs during recovery phase of repeat dose toxicity studies) (Heal et al., 2025).

Currently, *in vitro* assays for the detection of neurotoxicity are not widely used prior to performing animal studies in drug development. The neurite outgrowth assay that can be applied to rodent primary neurons or hiPSC-derived neurons is a useful tool to assess neuronal cell viability. Various endpoints can be measured, such as neurite length, cell body area, branch points and number of cell clusters (Bagheri et al., 2020; Zhang et al., 2022). This allows for a dynamic monitoring with automated image analysis.

But the next generation of *in vitro* assays in the neuroscience field is the human brain organoids (‘microbrain’ or ‘brain-on-chip’), which are 3D structures derived from hiPSCs reflecting human embryonic brain organization. They contain different cell types interacting together (neurons, glia) and have the advantage over animal-based models to better represent human brain function (Kim and Chang, 2023). Although their primary applications are for modelling neurodevelopmental or neurodegenerative disorders, they could also be used for assessing drug effects and hazard identification (Fan et al., 2022), or for personalized medicine if based on patient-derived cells (Giorgi et al., 2024). However, a recent EMA review of organ-on-chips used for medicines safety assessment showed that, while liver, kidney and heart are the most frequently modeled systems, CNS is less commonly used, both as single or multi-organ systems (Portela et al., 2026). This may be due to the current limitations and translational challenges of these models, namely their high variability and limited reproducibility, their structural and functional immaturity, and the lack of vascular and immune components (Park and Sun, 2025). Engineering strategies try to overcome these challenges (Fan et al., 2025).

In contrast with *in silico* proarrhythmia models that have been extensively developed over the past 10–12 years due to the CiPA paradigm and the integration of such models in the revised ICH S7B guidance, *in silico* tools to predict CNS side effects are less common and most efforts have focused on blood-brain barrier simulation models. In the framework of the former IMI2-NeuroDeRisk consortium mentioned earlier, a toolbox containing around 60 different *in silico* models based on various neurotoxicophores associated with seizure liability, suicidality, and peripheral neuropathies was established (Bryant et al., 2022). Some additional models linked to blood-brain barrier (for OATP2 and HCO antiporter) were also added. Any new chemical structure can be uploaded into the toolbox and tested against all models or a pre-selection of it. A heatmap is generated, which flags structure similarities with one or several of the toxicophores.

### 3.3 3Rs applied to respiratory safety pharmacology assessment

The respiratory system is the third vital system considered by the ICH S7A guidance. The latter considers that clinical observation is not adequate to assess respiratory function, and that respiratory rate and other relevant parameters of respiratory function (e.g., tidal volume or hemoglobin oxygen saturation) should be evaluated and quantified by using appropriate methodologies. However, the guidance does not provide any detail on the choice of optimal methodologies to use. Typically, respiratory safety pharmacology studies consist of plethysmography studies in rodents after single administration of the drug candidate (**Figure 4**). A recent multi-company retrospective analysis showed the poor predictive value of such preclinical studies to detect clinically relevant respiratory adverse events (Friedrichs et al., 2024). Alternative *in vivo* and *in vitro* approaches for respiratory assessment are given below.

#### 3.3.1 Reduction and refinement of *in vivo* respiratory assessments

Traditional respiratory assessments in rats—such as whole-body plethysmography (WBP) and head-out plethysmography—are limited by confinement, stress induction, and short recording durations, which can confound data interpretation (Coggins et al., 1981; Adler et al., 2004; Bohannon et al., 2016; Fares et al., 2019). To overcome these limitations, the DECRO system was developed: a miniaturized telemetry platform enabling continuous, non-invasive recording of respiration, ECG, and activity in freely moving, socially housed rats (Flenet et al., 2020; Fares et al., 2022). This innovation allows 24-hour and repeated recordings under home-cage conditions, capturing expected pharmacological effects (e.g., baclofen-induced respiratory depression) with high sensitivity and reproducibility, while also promoting Reduction and Refinement (Fares et al., 2022). Importantly, the system enables simultaneous respiratory and CNS functional assessments (Irwin test/FOB), demonstrated with baclofen, caffeine, and clonidine. This integration eliminates the need for separate studies, achieving a 100% reduction in animal use for dual endpoints (Fares and Champéroux, 2023). The inclusion of heart rate and locomotor activity further enhances interpretation of CNS, respiratory, and autonomic effects, and offers integrated insights into how drugs impact all these vital systems concurrently. Jacketed telemetry in rats offers a scientifically robust, ethically superior, and regulatory-compliant approach for modern safety pharmacology.

#### 3.3.2 Alternative *in vitro* approaches for respiratory function assessment

**Figure 5:**
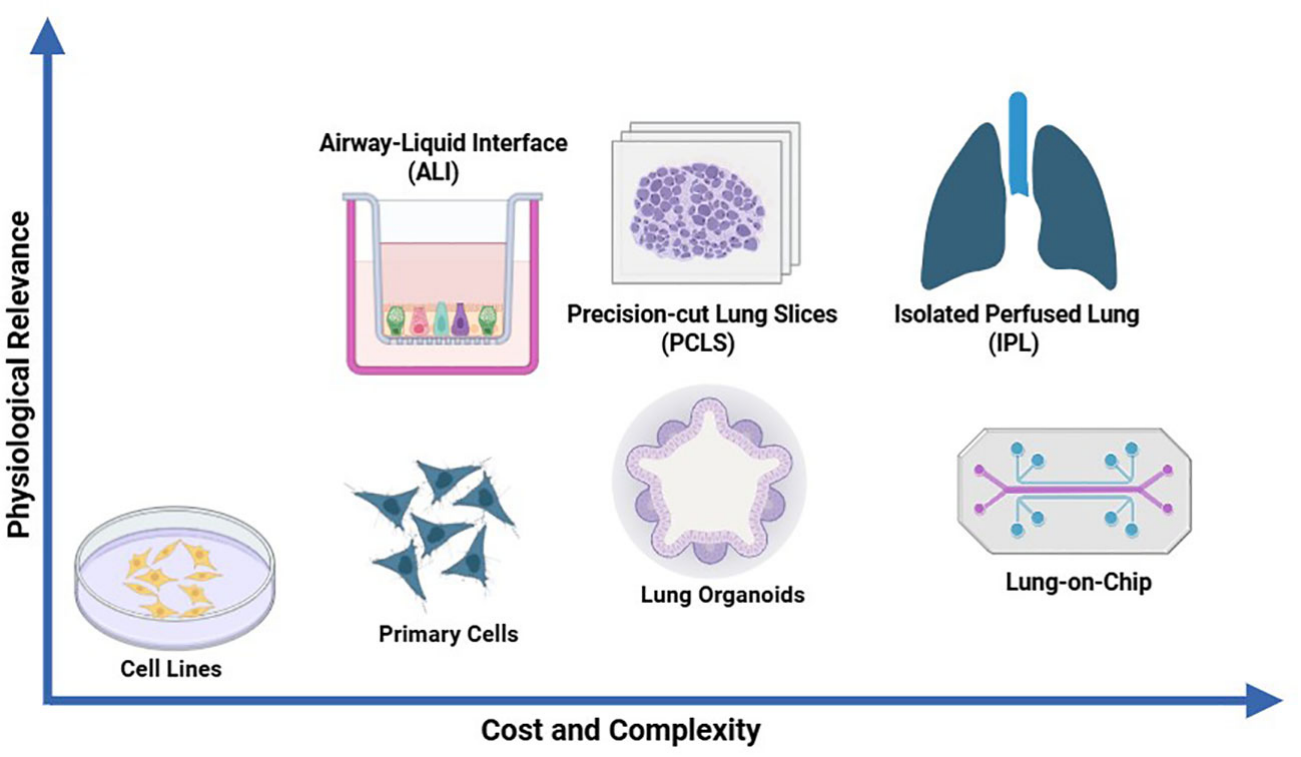
Alternative *in vitro* and *ex vivo* approaches for the assessment of drug-induced changes in respiratory function have the aim of creating human relevant systems that may replace animal use whilst balancing cost, complexity/laborious nature, clinically/physiological relevance and regulatory ambition. Created with BioRender.com.

A few advancing alternatives to *in vivo* respiratory studies are described hereafter. **Figure 5** illustrates that complex *in vitro* systems such as lung organoids are generally associated with higher costs compared to simple 2D cell models but have higher physiological relevance.

Traditional 2D monolayers *in vitro* models are useful for early screening, and the submerged cultures of the ciliated cells are used to check for structural and functional abnormalities. Nowadays there are numerous available cell lines with standardized and well-defined characteristics. Klein et al. (2013) published a list of the common lung and endothelial cell lines used for mono- and co-culture *in vitro* lung systems (Klein et al., 2013). As an alternative cell source, there are human pluripotent stems cells (hPSCs), which include embryonic stem cells (ESCs) and induced pluripotent stem cells (iPSCs). These 2D models are practical and inexpensive; however, they are only useful for yes/no approaches and do not allow for the details of response that have been reported as observed in 3D model systems, and pertinent to what would occur *in vivo*.

The Air-Liquid Interface (ALI) system was introduced by Witcuttj et al. in 1988 (Whitcutt et al., 1988). These ALI cultures offer a physiologically relevant *in vitro* model for safety pharmacology, particularly for inhalation toxicity studies, by allowing cells to differentiate (cilia, mucus, surfactant) with improved cell morphology and function, and by exposing them directly to aerosols/gases. Key advantages include: 1) a high physiological relevance by often using primary human cells, precision cut lung slices (PCLS), or human-derived bronchial epithelial cells (HBECs) for better mimicking the human lung than submerged cultures; 2) Direct exposure and accurate dosing, as the test articles can be applied directly to the apical surface, simulating *in vivo* inhalation exposure to aerosols, nanoparticles, and gases; 3) act as an intermediate step between standard *in vitro* assays and *in vivo* studies,. While disadvantages of these ALI cultures include high technical complexity as these require specialized instrumentation, high cost, high variability, labor-intensive, limited contribution of immune system, no circulation, low-throughput, and requiring 3–4 weeks for differentiation.

The precision cut lung slices (PCLS) are an *ex vivo* method, offering a 3D model that preserves native lung architecture and cell-type diversity (epithelium, smooth muscle, macrophages), using tissue already obtained and reliably stored. These PCLS can be obtained from either murine tissue or human tissue (healthy or diseased), reducing animal use while enhancing translational accuracy, and have been known to constrict when exposed to various stimuli, but their responses reduce over time (Neuhaus et al., 2017). These sections however only give a “snapshot” of what the lung looks like at the exact moment of fixation (Liu et al., 2019), and lack intact blood flow, circulating immune cells, and neural innervation. This would therefore only give an indication of what a potential “whole system” response may be and thus can be considered as a scientific ‘middle ground’ between established *in vitro* models and *in vivo* models.

Lung organoids are derived by self-organized primary lineages, such as ESC, iPSC, or organ stem/progenitor derivatives that develop into complex airway or alveolar tissues (Lancaster and Knoblich, 2014), offering a more integrated model and bridging the gap between traditional 2D cell cultures and animal models, particularly for identifying tissue-specific safety assessments. The limited number of cells needed to create an organoid further makes them potentially favorable, due to shortage of established primary cell lines. However, they are often used as complementary tools rather than a complete replacement for *in vivo* studies due to the lack of systemic, vascular, and immune components.

Lung-on-a-chip (LoC) technology was developed by the Wyss Institute founding director, Dr. Don Ingber at Harvard University (Huh et al., 2012). In 2013, he received the NC3Rs 3Rs Prize for his innovative LoC, a microdevice lined by human cells that recapitulates complex functions of the living lung. Indeed, this micro-engineered and microfluidic device is closely mimicking human lung tissue architecture, featuring alveolar-capillary barriers with epithelial and endothelial cells. This can simulate the physical forces of breathing (stretching), fluid flow (shear stress), and air exposure, enabling dynamic assessment of inhaled substances. It is a better replicate structure and function of the human lungs, including fluidics at a much smaller scale. These chips can be customized using patient-derived cells (e.g., in cystic fibrosis models) to test drug responses for specific individuals. While LoC technologies are revolutionizing safety pharmacology with their human-like and dynamic environments, they face limitations in scalability and cost that need to be addressed for broader adoption.

In summary, respiratory *in vitro* platforms are generally used for mechanistic understanding, but more rarely for drug safety screening and for clinical decision−making. Moreover, quantitative metrics on sensitivity/specificity of these assays are rarely (if ever) reported.

### 3.4 3Rs applied to gastrointestinal function assessment

The assessment of the gastrointestinal (GI) function is not part of the ICH S7A core battery but is recommended based on the knowledge obtained on the drug under investigation (e.g., primary or secondary pharmacology profile), when GI adverse effects are suspected. Although they rarely lead to drug discontinuation and are generally not life-threatening, GI side effects such as diarrhea/constipation or nausea/vomiting, can affect the quality of life, in particular after chronic treatment. The GI tract is also the primary site of drug absorption, making its functional assessment critical in early drug development. Traditionally, safety pharmacology studies evaluating GI function mainly consisted of charcoal meal test in rodent species, to measure gastric emptying and intestinal transit. Various *ex vivo* or *in vitro* models already exist, and have been used for many years, usually based on fresh animal isolated segments of intestine, such as the guinea pig ileon (Bertaccini et al., 1979). However, they still require euthanizing the animal to collect the tissues. Moreover, the anatomy, physiology and biochemistry of the GI tract significantly differ between species. Therefore, animal-based models, while informative, may fail to replicate human-specific GI physiology. Human-based systems are also extensively used, such as Caco-2 cells, or the famous Ussing chamber, invented in 1946. It consists of two fluid-filled, heated, gassed chambers, separated only by mucosal tissue preparation (intestinal or respiratory). It is considered the gold standard to evaluate drug absorption, transport, and metabolism (Clarke, 2009). Nowadays, more advanced human-based *in vitro* models have emerged as promising alternatives for GI safety evaluation or for DPMK research.

#### 3.4.1 Novel *in vitro* GI models

Recent developments include 2D and 3D cell culture systems, organoids, and organ-on-chip technologies. Organoids, derived from intestinal stem cells, self-organize into multicellular structures mimicking villus and crypt domains, enabling the study of epithelial responses to drugs. These models can be tailored to represent healthy or diseased states, enhancing their translational relevance (Peters et al., 2020).

Microphysiological systems (MPS), such as gut-on-a-chip platforms, incorporate fluid flow and mechanical strain to simulate peristalsis and nutrient transport. These systems support co-culture of epithelial, immune, and microbial cells, allowing for integrated assessment of drug-induced GI toxicity, including inflammation, barrier disruption, and microbiome interactions (Neto et al., 2025).

*In vitro* digestive simulation models also play a role in evaluating drug absorption and metabolism. These range from static single-compartment setups to dynamic multi-compartment systems that mimic physiological digestion processes, offering reproducible and ethically sound alternatives to animal testing (Li et al., 2024).

Within safety pharmacology, *in vitro* GI models are primarily used to identify and rank compounds with GI liability, with diarrheagenic risk emerging as a key application (Peters et al., 2020; Debad et al., 2025). Human 3D GI microtissue assays that measure transepithelial electrical resistance (TEER) as a readout of tight junction integrity can discriminate drugs that cause clinical diarrhea from those that do not, including cases where short-term animal studies failed to predict risk. TEER and related barrier endpoints therefore serve as important mechanistic surrogates for epithelial injury (Peters et al., 2019).

High-throughput 2D assays using primary human intestinal stem cell–derived monolayers extend this approach by integrating TEER with cell viability (e.g., nuclear counts) and proliferation (e.g., EdU incorporation) across multiple donors. These multi-donor designs enhance population-level relevance and improve prediction accuracy for clinical diarrhea (Pike et al., 2025). Gut-on-chip platforms and organoid–immune co-cultures provide additional mechanistic depth, allowing assessment of cytokine release, immune cell recruitment, and microbiota-modulated injury—features especially relevant for inflammation-driven GI toxicity (Ashammakhi et al., 2020; Macedo et al., 2023; Ofori‐Kwafo et al., 2025). Collectively, these *in vitro* systems support compound triage, medicinal chemistry optimization, and dose/regimen refinement by generating human-relevant GI safety data early in development, while reducing reliance on exploratory animal studies.

Advanced primary or organoid-derived intestinal monolayers, often combined with region-specific media and transporter/enzyme profiling, are increasingly used to refine segmental absorption estimates, assess drug–drug interactions at intestinal transporters, and characterize metabolism by CYPs and other enzymes (Fedi et al., 2021; Debad et al., 2025). Static and dynamic *in vitro* digestion models simulate dissolution, solubilization, and precipitation of oral formulations under physiologic pH and bile conditions, providing inputs for absorption modeling and supporting biopharmaceutics risk assessments, particularly for poorly soluble compounds (Xavier et al., 2023). Integration with endothelial cells, hepatocytes, and biliary-like cells in multi-organ MPS enables evaluation of sequential intestinal absorption, hepatic metabolism, and biliary excretion, capturing first-pass and enterohepatic processes in a more physiologically connected manner. Collectively, these DMPK-oriented *in vitro* methods complement GI safety-focused assays by describing how drugs and formulations behave along the GI tract, thereby strengthening mechanistic understanding of exposure–response relationships and informing dose selection, formulation design, and clinical study planning.

Building on these advances, several opportunities could increase the regulatory relevance of *in vitro* GI models as complements—or, in some contexts, alternatives—to the *in vivo* endpoints described in ICH S7A. Expanding beyond the current focus on small intestine and colon toward robust, standardized models of the stomach, duodenum, jejunum, and ileum would improve coverage of region-specific physiology, including differential pH, enzyme expression, bile exposure, and microbial load.

Currently, *in vitro* efforts emphasize GI injury and diarrheagenic risk, increasingly addressed through TEER-based barrier assays and cytotoxicity measures. Future work could focus on defining and qualifying formal surrogate endpoints for the broader set of S7A parameters, including gastric and bile secretion, transit time, and ileal contraction. Dynamic digestion models that approximate gastric secretion and pH, coupled to intestinal epithelia, could be further developed and standardized as functional analogues of classical *in vivo* secretory measurements. Similarly, MPS platforms that introduce cyclic strain and fluid flow to emulate peristalsis could be refined to better capture neuromuscular activity and motility patterns, enabling more direct mechanistic assessment of ileal contraction and transit.

As the field matures through systematic benchmarking of *in vitro* endpoints against clinical data and *in vivo* S7A-type measures these models have the potential to more directly address guideline expectations, support risk-based interpretation of GI findings, and ultimately reduce reliance on dedicated animal studies in GI safety pharmacology.

#### 3.4.2 Refining *in vivo* methodologies for GI function assessment

In parallel to the use of these *in vitro* systems and in alignment with the Refinement principle, minimally-invasive systems have been developed to measure gastrointestinal motility and other parameters such as gastric pH or intra-abdominal pressure. The SmartPill™ is a multisensory ingestible capsule well-known in clinical setting to monitor transit time, pH, pressure and temperature. It has also been successively used in minipigs (Knibbe et al., 2023) and dogs both for veterinary medicine purposes (Warrit et al., 2017) or preclinical research (Koziolek et al., 2019). Another example of wireless system is the Bravo™ pill used for esophageal pH test in patients. It has also been applied in various animal species (rats, dogs, monkeys) (Rudholm et al., 2008; Kook et al., 2014) for ambulatory esophageal pH monitoring or to assess the influence of pH on drug absorption and pharmacokinetics. A recent technology developed in rats is an ingestible capsule for non-invasive optical gut stimulation (ICOPS) (Elsherif et al., 2025). This small capsule of less than 2 cm length and 0.22 g weight is equipped with micro-LED lights and electronics for wireless powering based on the inductive coupling and is safely excreted in feces within 20h. It allows optogenetic stimulation of the enteric nervous system to better understand the gut-brain axis. However, such sophisticated technique has never been applied in safety pharmacology so far.

### 3.5 3Rs applied to renal function assessment

As for the GI system, studies of the renal function are considered as supplemental studies by the ICH S7A guidance, to be conducted when there is a cause for concern identified from primary or secondary pharmacology profile or based on renal findings in early toxicology studies. Repeat dose toxicity studies generally include by default some renal or urinary parameters such as urinary volume, osmolality, pH, fluid/electrolyte balance, proteins, cytology, or blood chemistry determinations such as blood urea nitrogen, creatinine and plasma proteins. Seven drug-induced kidney injury (DIKI) biomarkers (among which KIM-1 and clusterin) have also been qualified by regulators for nephrotoxicity assessment in the rat but are not yet routinely included in toxicology studies. Dedicated safety pharmacology studies to assess renal function are also rarely conducted, as highlighted by previous surveys across 15 top pharmaceutical companies (Benjamin et al., 2015).

#### 3.5.1 Refining *in vivo* methodologies for renal function assessment

When conducted, in most cases, *in vivo* renal safety pharmacology studies mostly include the measurement of the glomerular filtration rate (GFR) in rodents, which requires the use of a tracer to determine either its excretion rate in urine or its elimination kinetics from plasma. Urine sampling generally requires singly housing the animals in metabolic cages, while blood sampling necessitates handling and restraint, which are stressful (Passini et al., 2025).

Refinement approaches may include automated blood sampling techniques, which allow blood collection from freely moving rodents even during night period (Han et al., 2013), or microsampling, which reduces the volume of blood required to just a few microliters. Such systems can of course be used not only for renal function assessment but for any studies requiring repeated blood sampling (PK, pharmacology, etc…) (Hopper, 2016; Prior et al., 2025). Another example of refinement is the use of hydrophobic sand for urine collection in rodents. This method proved to be efficient in maintaining volume and integrity of urine samples, while decreasing stress related to single housing in metabolic cages and the risk of injury (Hoffman et al., 2018) and can be applied for urine collection in all toxicology studies. An innovative 3D-printed two-compartmental device was also designed to allow urine collection from two home-caged mice keeping social interactions (Douté et al., 2024).

#### 3.5.2 Novel *in vitro* renal models

Over the last decade, significant progress has been made in the development of human-relevant kidney models that may reduce the need for animal testing. Two-dimensional renal proximal tubule epithelial cell cultures (RPTEC) enable high-throughput screening of nephrotoxicity by measuring mechanistic endpoints such as viability, oxidative stress, and specific injury biomarkers including KIM-1 and NGAL. However, these cultures lack complex architecture and transporter expression, limiting physiological relevance. To address these gaps, 3D spheroids and organoids derived from either human proximal tubule cells (Sakolish et al., 2023; Arakawa et al., 2024), urine-derived stem cells (Guo et al., 2020; Yu et al., 2023), or pluripotent stem cells have been developed, demonstrating improved expression of drug transporters (e.g., OAT1) and enhanced sensitivity to nephrotoxic compounds like cisplatin and amphotericin B, when assessed via ATP depletion, KIM-1 measurement, CYP induction, and structural injury markers. Furthermore, a ‘kidney-on-a-chip’ technology was developed, which incorporates perfusable architecture, co-cultures (e.g., RPTECs and endothelial cells), and functional readouts such as glucose reabsorption and TEER (Huang et al., 2024; Guimaraes et al., 2025). These platforms improve transporter activity, injury biomarker detection, and organ-level responses compared to static 2D cultures. This tiered strategy—from 2D assays to 3D and microfluidic systems—enables more human-relevant, mechanistic assessment of nephrotoxicity while reducing reliance on animal models. However, significant challenges remain, including immaturity, poor vascularization, or absence of urinary excretion, before being able to routinely use such *in vitro* systems for safety testing (Xi and Song, 2025).

## 4 Role and contribution of the SPS in 3Rs

**Figure 6:**
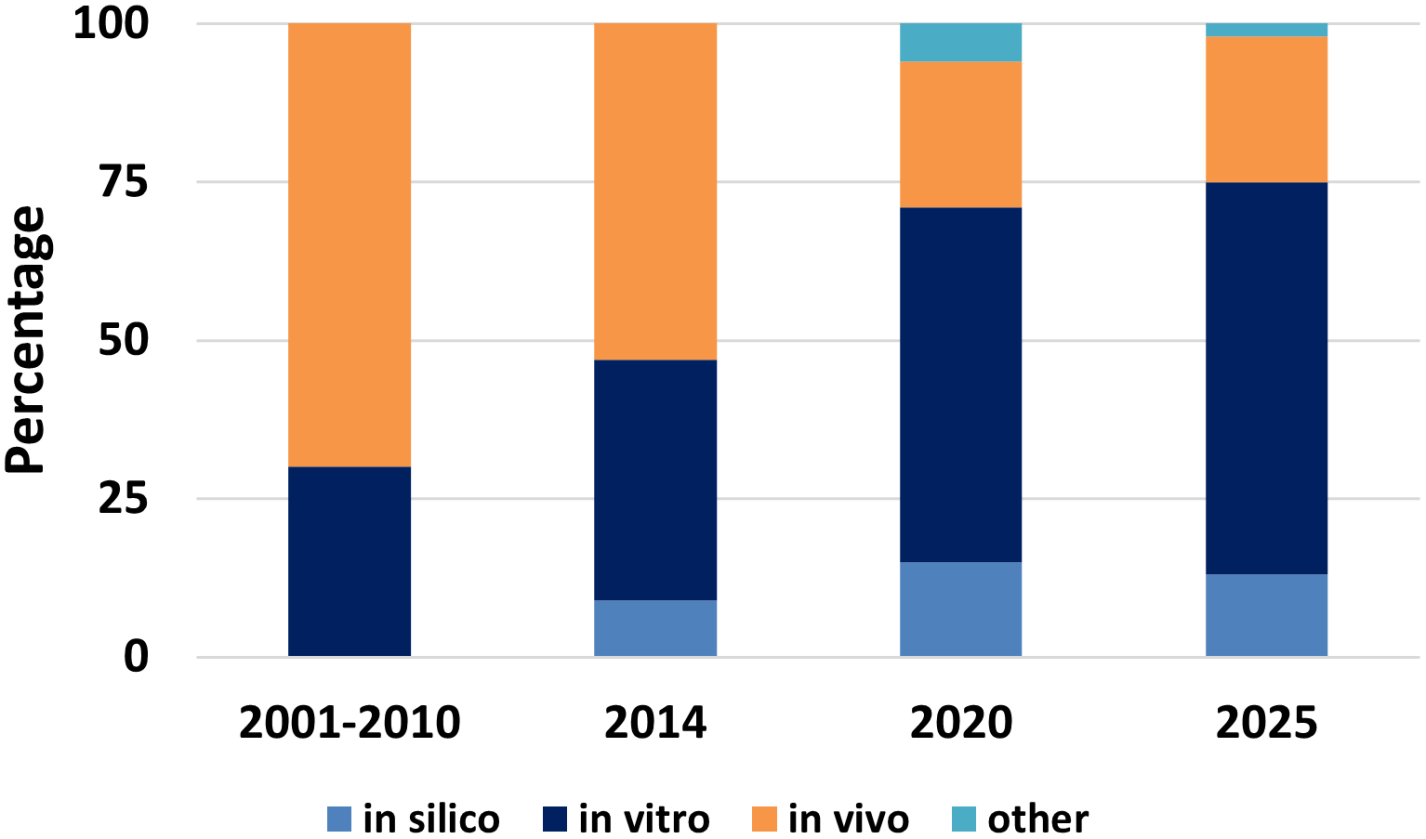
Temporal evolution of the poster categories presented at the Safety Pharmacology Society (SPS) annual meetings. A regular increase in the percentage of *in silico* and *in vitro* topics is noted. Adapted from (Valentin et al., 2023).

The Safety Pharmacology Society (SPS) was founded as a non-profit organization in August 2000, just after the adoption of the ICH S7A guidance. Its mission is to promote knowledge, development, application, and training in safety pharmacology. Although the application and promotion of the 3Rs principles have always been an essential aspect of the SPS mission and vision, no official committee or working group on this topic originally existed within the Society. Over years, the number of 3R-related topics (in particular *in silico* and *in vitro* assays) presented during SPS annual meetings has constantly increased (**Figure 6**), showing the growing interest of the safety pharmacology community for the development and validation of innovative approaches and the willingness to reduce, refine, and replace *in vivo* methods. The idea of creating a dedicated SPS 3Rs working group (SPS 3Rs WG) first emerged on the SPS on-line community in March 2023. The official creation of the 3Rs WG was approved by the SPS Board of Directors in October 2023, a Chair was appointed (Dr. K. Derakhchan) and the first meeting was organized in February 2024, with more than 40 members/volunteers from USA, Canada, Europe and Japan.

The vision of the group is to serve the SPS and its members by championing 3Rs in safety pharmacology to support research, testing and development of safe drugs.

The mission of this group was defined as follows:

- Support and promote the development and implementation of predictive/translational novel approach methodologies/technologies as alternatives to the use of animals in safety pharmacology
- Where animal use is necessary, advocate for optimized model selection, refined endpoints and study design, to quantitatively predict human risk while improving animal welfare
- Support the validation and qualification of models in alignment with regulatory guidelines (ICH S7) to provide human relevant risk assessment and broader application
- Support the adoption of 3Rs across the safety pharmacology community by collaboration and sharing of scientific advancement and technology in the discovery and development of new medicines
- Provide training and education opportunities on 3Rs applications in safety pharmacology
- Recognize high impact 3Rs publications or projects
- Serve as an SPS ambassador in the 3Rs/NAMs community outside of the society.

During the 2025 SPS Annual Meeting, a competition for the Best 3Rs Abstract Awards was organized for the first time. About 40% of the abstracts submitted participated in the competition, showing once again the interest of the safety pharmacology community for the 3Rs principles and the development of NAMs.

## 5 Future perspectives

Recently, Valentin and Leishman (Valentin and Leishman, 2023) critically examined the ICH S7A guideline on safety pharmacology, originally released in 2000, and advocated for its revision. While the guideline has largely fulfilled its goal of protecting clinical trial participants from adverse drug effects, the safety-related attrition and post-approval withdrawals remain high, showing its limitations. The authors argue that scientific, technological, and regulatory advancements over the past two decades necessitate a modernized, integrated, and sustainable approach. They emphasize the need to align the guidelines with evolving drug development paradigms, including novel therapeutic modalities (e.g., RNA therapeutics, gene therapies) and better integration of NAMs and 3Rs principles. Indeed, the current S7A guideline mainly relies on *in vivo* models, with only limited use of *in vitro*/in silico methods. While the CiPA initiative has advanced cardiovascular safety assessment, no comparable approach has been established for the other systems. Six main pillars have been identified for consideration to revise ICH S7A (Valentin and Leishman, 2025) of which at least three are directly linked to the 3Rs: integrating human-relevant *in vitro/in silico* models, leveraging state-of-the-art *in vivo* platforms, and establishing validation principles for novel assays. Those recommendations were largely supported by the broader industry community (Valentin et al., 2025b). At this stage, a revision procedure under ICH processes will be proposed, starting with the preparation of a concept paper outline to initiate formal discussions for endorsement by the ICH management committee. Although this revision process may take years, much work has been agreed upfront through formal discussions with relevant stakeholders which it is believed would speed up the revision process, and it is anticipated the revision will certainly change the future of safety pharmacology.

As highlighted in this review, the principles of the 3Rs have been central to safety pharmacology since its inception, exemplified by successful innovations such as the adoption of telemetry technology and the use of hiPSC-derived assays for cardiovascular and CNS safety assessment. Pharmaceutical industry, service providers, technology developers, and regulators have already started to embrace the paradigm change as shown by the increasing number of publications, consortia or working groups actively involved in the NAMs field for safety assessment (Beilmann et al., 2025; Shenton et al., 2025). The journey is far from over, but there are great new opportunities ahead.
